# Molecular Glues: The Adhesive Connecting Targeted Protein Degradation to the Clinic

**DOI:** 10.1021/acs.biochem.2c00245

**Published:** 2022-07-20

**Authors:** Janet M. Sasso, Rumiana Tenchov, DaSheng Wang, Linda S. Johnson, Xinmei Wang, Qiongqiong Angela Zhou

**Affiliations:** CAS, a division of the American Chemical Society, 2540 Olentangy River Road, Columbus, Ohio 43202, United States

## Abstract

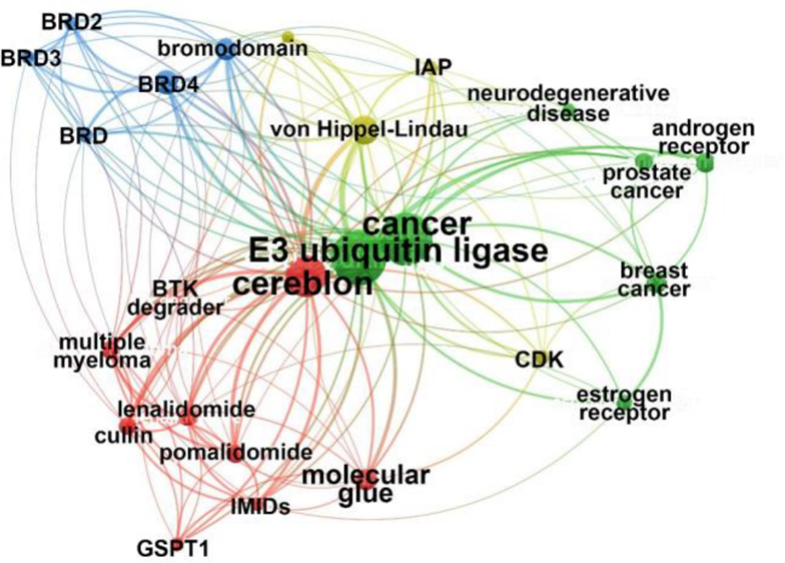

Targeted protein degradation is a rapidly exploding drug discovery strategy that uses small molecules to recruit disease-causing proteins for rapid destruction mainly via the ubiquitin–proteasome pathway. It shows great potential for treating diseases such as cancer and infectious, inflammatory, and neurodegenerative diseases, especially for those with “undruggable” pathogenic protein targets. With the recent rise of the “molecular glue” type of protein degraders, which tighten and simplify the connection of an E3 ligase with a disease-causing protein for ubiquitination and subsequent degradation, new therapies for unmet medical needs are being designed and developed. Here we use data from the CAS Content Collection and the publication landscape of recent research on targeted protein degraders to provide insights into these molecules, with a special focus on molecular glues. We also outline the advantages of the molecular glues and summarize the advances in drug discovery practices for molecular glue degraders. We further provide a thorough review of drug candidates in targeted protein degradation through E3 ligase recruitment. Finally, we highlight the progression of molecular glues in drug discovery pipelines and their targeted diseases. Overall, our paper provides a comprehensive reference to support the future development of molecular glues in medicine.

Currently, targeted protein degradation (TPD) has become a groundbreaking strategy in drug discovery. This approach is emerging as a novel therapeutic method for aiming at diseases such as cancer, inflammatory and immune diseases, and infections, as many of them are driven by the aberrant expression of a pathogenic protein.^1−5^ Recent research using TPD has focused on recruiting disease-causing proteins previously thought to be “undruggable” due to their lack of canonical ligand binding sites for rapid destruction and elimination via the ubiquitin–proteasome pathway. The ubiquitin–proteasome system (UPS) is a major mechanism for cellular protein degradation and maintaining protein homeostasis, as part of the regular cellular housekeeping processes. Thus, the potential breadth of TPD applications is almost unlimited.

The UPS process involves an enzyme cascade that results in ubiquitination of the protein of interest (POI). Ubiquitination is at the heart of both proteasomal and autophagy-mediated protein degradation, with E3 ligases as the critical components of the ubiquitination cascade.^6,7^ Of the more than 600 E3 ubiquitin ligases encoded by the human genome, only a few have been exploited for targeted protein degradation, for example, cereblon (CRBN), VHL, MDM2, DDB1, DCAF15, and SCF βTRCP. These subunits can be targeted by degraders that cause conformational changes that promote the formation of a ternary complex with the POI.^8,9^ In principle, the formation of a ternary complex induces molecular proximity between the catalytic site of the E3 ligase and the POI, prompting ubiquitin transfer and subsequent proteasomal degradation of the POI. Identifying successful strategies for discovering ligands that bind to E3 ligases becomes an attractive and exciting research objective.^2,10,11^

Compared to traditional pharmacological target protein inhibition, protein degradation offers two crucial advantages. First, targeted degradation is a catalytic process because degraders act via transient binding rather than competitive occupancy and successfully dissociate after promoting polyubiquitination of the disease-causing protein.^8,12^ As such, a single degrader can destroy many copies of a pathogenic protein, thereby providing a greater efficiency at very small doses. Second, while protein inhibitors block the active site of a pathogenic protein, degraders ablate all of its functions, providing higher sensitivity to drug-resistant targets and a better chance to affect nonenzymatic protein functions.^13−15^

Many key discoveries have contributed to advancing the targeted protein degradation notion as we know it today. The earliest-known published description of the concept of chimeric degraders is in a patent filed in 1999 by a biotechnology company, Proteinix, proposing taking over the cellular protein degradation system (Figure 1).^16^ The concept of utilizing the ubiquitin-driven natural protein degradation system for therapeutic purposes focused on designing small molecules that recruit E3 ligases for degradation of a POI (Table 1). In 2001, the first *in vitro* proof-of-concept study was published, demonstrating that a peptide-based protein-targeting chimeric molecule, Protac-1, recruiting E3 ligase β-TRCP, successfully led to the degradation of a cancer-associated protein, MetAP2; thus, the name PROTAC (proteolysis-targeting chimera) was introduced.^17^ Later, the finding of a peptide from HIF1α, which binds the VHL E3 ligase, resulted in the construction of cell-penetrating PROTACs, which degraded a variety of proteins (Table 1).^17,18^ As indicated, these early PROTACs contained peptide ligands for the E3 ligase; a report of a canonical small molecule PROTAC, an androgen receptor (AR) degrader using nutlin-3 for recruiting of MDM2, was published in 2008.^19^ This, and the later discovery of small molecule mimetics of the HIF1α peptide,^20−22^ expedited the rational design of small molecular PROTACs.^21−25^

**Figure 1:**
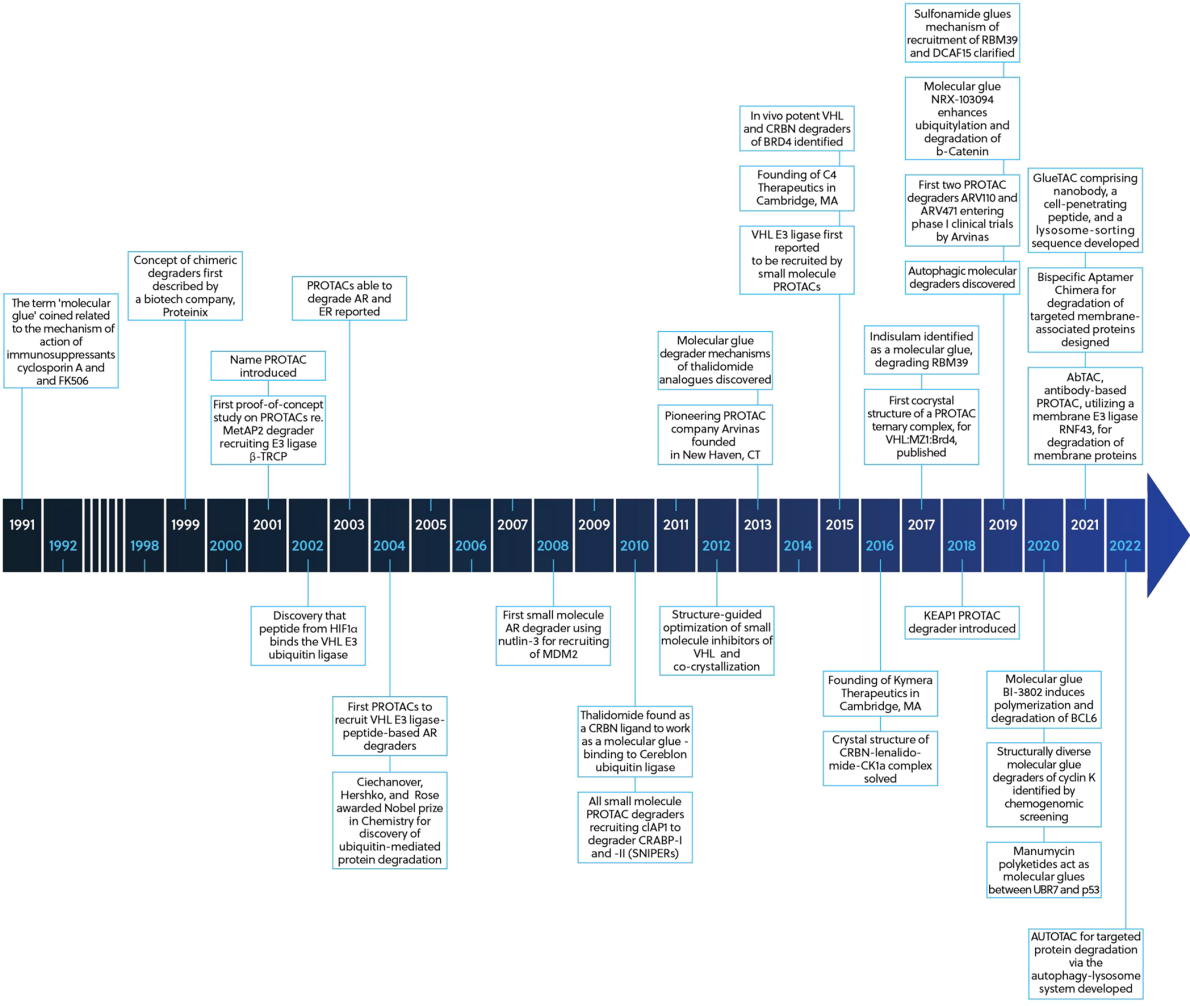
Timeline of major targeted protein degrader research and development milestones.

**Table 1:** Exemplary Proteins Successfully Targeted for E3 Ligase Degradation

targeted protein	E3 ligase/subunit recruited	degrader	year
MetAP2^17^	βTRCP	PROTAC	2001
androgen receptor^34^	βTRCP	PROTAC	2003
estrogen receptor^34^	βTRCP	PROTAC	2003
androgen receptor^18^	VHL	PROTAC	2004
aryl hydrocarbon receptor^35^	VHL	PROTAC	2007
androgen receptor^19^	MDM2	PROTAC	2008
estrogen receptor^36^	VHL	PROTAC	2008
FRS2α^37^	VHL	PROTAC	2008
PI3K^37^	VHL	PROTAC	2008
CRABPI and CRABPII^23^	cIAP	PROTAC	2010
RAR^23^	cIAP	PROTAC	2010
androgen receptor^38^	cIAP	SNIPER	2011
estrogen receptor^38^	cIAP	SNIPER	2011
TACC3^39^	cIAP	SNIPER	2014
BET (BRD2, BRD3, and BRD4)^22,40^	VHL	PROTAC	2015
BET (BRD2, BRD3, and BRD4)^41,42^	CRBN	PROTAC	2015
ERRα^21^	VHL	PROTAC	2015
FKBP12^41^	CRBN	molecular glue, PROTAC	2015
RIPK2^21^	VHL	PROTAC	2015
AKT^43^	VHL	PROTAC	2016
BCR–ABL^44^	VHL	PROTAC	2016
BCR–ABL^44^	CRBN	PROTAC	2016
tau^45^	VHL	PROTAC	2016
RBM39^46^	DCAF15	molecular glue	2017
RBM23^47,48^	DCAF15	molecular glue	2019

The establishment of the PROTAC strategy was further augmented by the finding of degrader compounds that became known as molecular glues. Molecular glues^26,27^ are monovalent small molecules (<500 Da) that reshape the surface of an E3 ligase receptor, promoting novel protein–protein interactions (PPIs). In contrast to the original PROTACs, in which two ligands are connected by a flexible linker that can twist and turn and allow the two proteins to form contacts, molecular glues were believed to more directly enhance complex formation between an E3 ligase and a target protein by squeezing between protein–protein interfaces and are generally defined as small molecules that interact with two protein surfaces to induce or enhance the affinity of these two proteins for each other (Figure 2).^28^ The term “molecular glue” was initially coined to describe the mechanism of action of cyclosporin A and FK506 inducing novel protein–protein associations.^29^ The molecular glue degraders such as thalidomide were discovered retrospectively, after their FDA approval and later detection of their immunomodulatory and anti-inflammatory activity.^30^ The E3 ligase cereblon was identified as the target of thalidomide and its analogues, lenalidomide and pomalidomide, known as immunomodulatory imide drugs (IMiDs), with reference to cancer therapy.^30^ They are some of the founding examples of molecular glues for targeted degradation. In fact, recent structural and biophysical data have shown that PROTACs can function in the same way as molecular glues, inducing neo-PPIs between the E3 ligase and the target protein and thus contributing to the formation of stable ternary complexes between the neo-substrate, PROTAC, and E3 ligase.^31,32^ In this way, the distinction between PROTACs and molecular glues becomes difficult to define. Moreover, as recently reported, simple structural modifications may easily convert a bona fide MDM2 PROTAC degrader into a molecular glue.^33^

**Figure 2:**
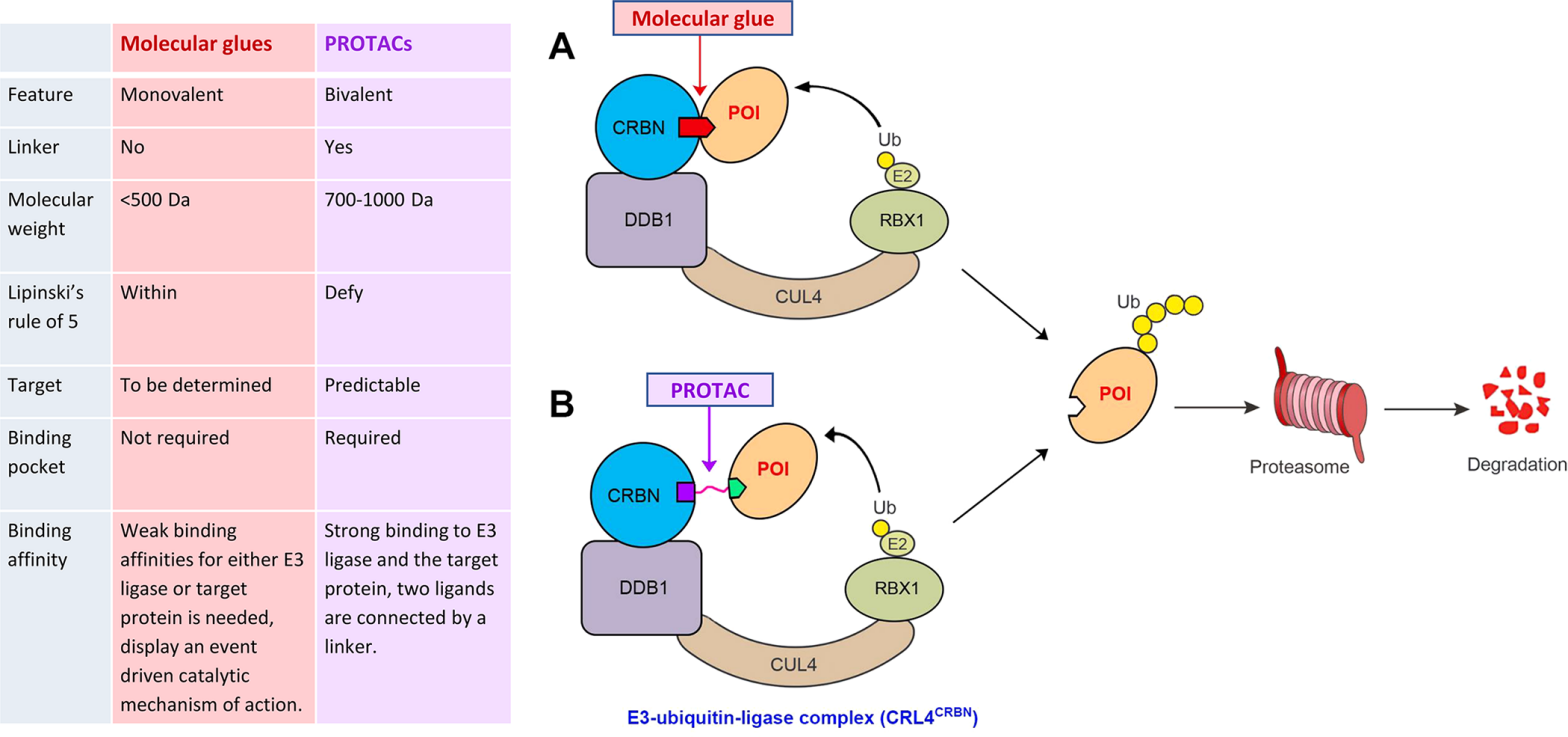
Schematic presentation of the degradation of a protein of interest (POI) via the ubiquitin (Ub)–proteasome system using (A) a molecular glue or (B) a PROTAC bound to the E3 ubiquitin ligase CUL4–RBX1–DDB1–CRBN (CRL4CRBN) complex.

The effects of protein–protein interaction in ternary complex formation have been characterized by a cooperativity term.^31,49,50^ It is defined as the ratio of the dissociation constants for the interactions between the ligand and one of the two protein components in the absence and presence of the other. The cooperativity of a molecular glue system is physically determined by the complementary interface between the ligand and the dimerization partner as successfully estimated by crystal structure studies.^51^ Thus, for molecular glues, cooperativity is a key parameter describing the activity of the compounds, which informs the competence of a molecular glue compound.^51^

The first rational discovery of molecular glues between a ligase and a protein of interest involved a series of compounds that enhance the interaction between an oncogenic transcription factor, β-catenin, and its cognate E3 ligase, SCF β-TrCP.^52^ These compounds promote the ubiquitination of β-catenin by β-TrCP and induce further degradation of β-catenin in cells. In addition to E3 ubiquitin ligase-based molecular glues, there are other molecular glues that induce protein degradation and/or dysfunction through various mechanisms of action, including autophagy-mediated protein degradation, MEK subcomplex stabilization, KRAS mutant inhibition, α-tubulin polymerization stabilization, FK506 binding protein 12 (FKBP12) protein degradation, etc.^4^ Recently, a new approach has been applied to a challenging target class, the intrinsically disordered proteins. It involves forcing disordered proteins to acquire a druggable interface using molecular glues to stabilize their interaction with 14–3–3 adaptor proteins, a signaling hub for critical cell processes.^53^ These examples demonstrate that molecular glues are emerging as a promising new therapeutic strategy.

Molecular glues are expected to have pharmacological properties that are better than those of PROTACs (Figure 2). In contrast to PROTACs, they are much smaller and thus more easily abide by Lipinski’s rule of five for drug conformity, which suggests an upper limit of molecular properties expected to enhance the probability for good oral bioavailability.^54^ Because they are smaller, they are expected to have higher membrane permeability and better cellular uptake and in general are less likely than PROTACs to pose a significant challenge for penetration of the blood–brain barrier, an important requirement for treating central nervous system (CNS) disorders.^55^ Small molecule glues have also been shown to be able to reprogram the binding partners of scaffolding proteins or to enhance the endogenous interaction between two proteins.^26^ On the contrary, though, an important advantage of PROTACs is their versatility; they allow for modular design to rapidly connect one enzyme with many targets. Thus, PROTACs are relatively easy to design and the target proteins are predictable.^56^ Although a simple modular approach to the design of molecular glues is not possible, recent advances in rationally designing molecules that optimize the cooperativity of binding of two proteins in a ternary complex has been successful.

To shed light on the advances in targeted protein degradation research, here we examine the publication landscape, analyze the relevant data from the CAS Content Collection,^57^ and thoroughly review both molecular glue drug candidates in targeted protein degradation through E3 ligase recruitment and the development of molecular glues in medicinal chemistry and drug discovery.

## Landscape of Research Publications from the CAS Content Collection Related to Targeted Protein Degraders

**Figure 3:**
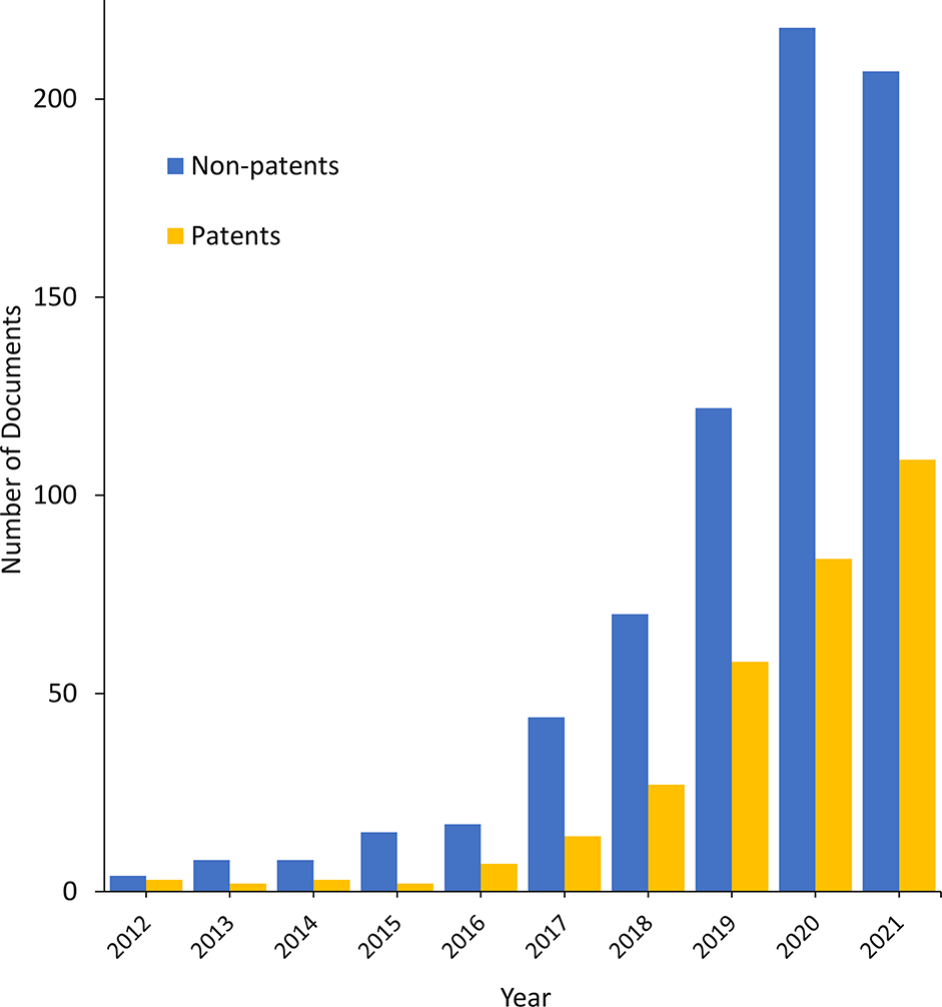
Trends in the number of publications related to protein degraders in the past decade, including journal articles and patents.

The CAS Content Collection^57^ represents the largest human-curated collection of published scientific knowledge. It is particularly useful for quantitative analysis of global scientific publications against variables such as time, research area, formulation, application, disease association, and chemical composition. Currently, there are more than 1000 targeted protein degrader (TPD)-related publications in the CAS Content Collection, including mainly journal articles and patents. Figure 3 illustrates trends in the number of publications over time, showing the explosive growth in recent years, from single digits in 2014 to hundreds of publications in the past two to three years.

**Figure 4:**
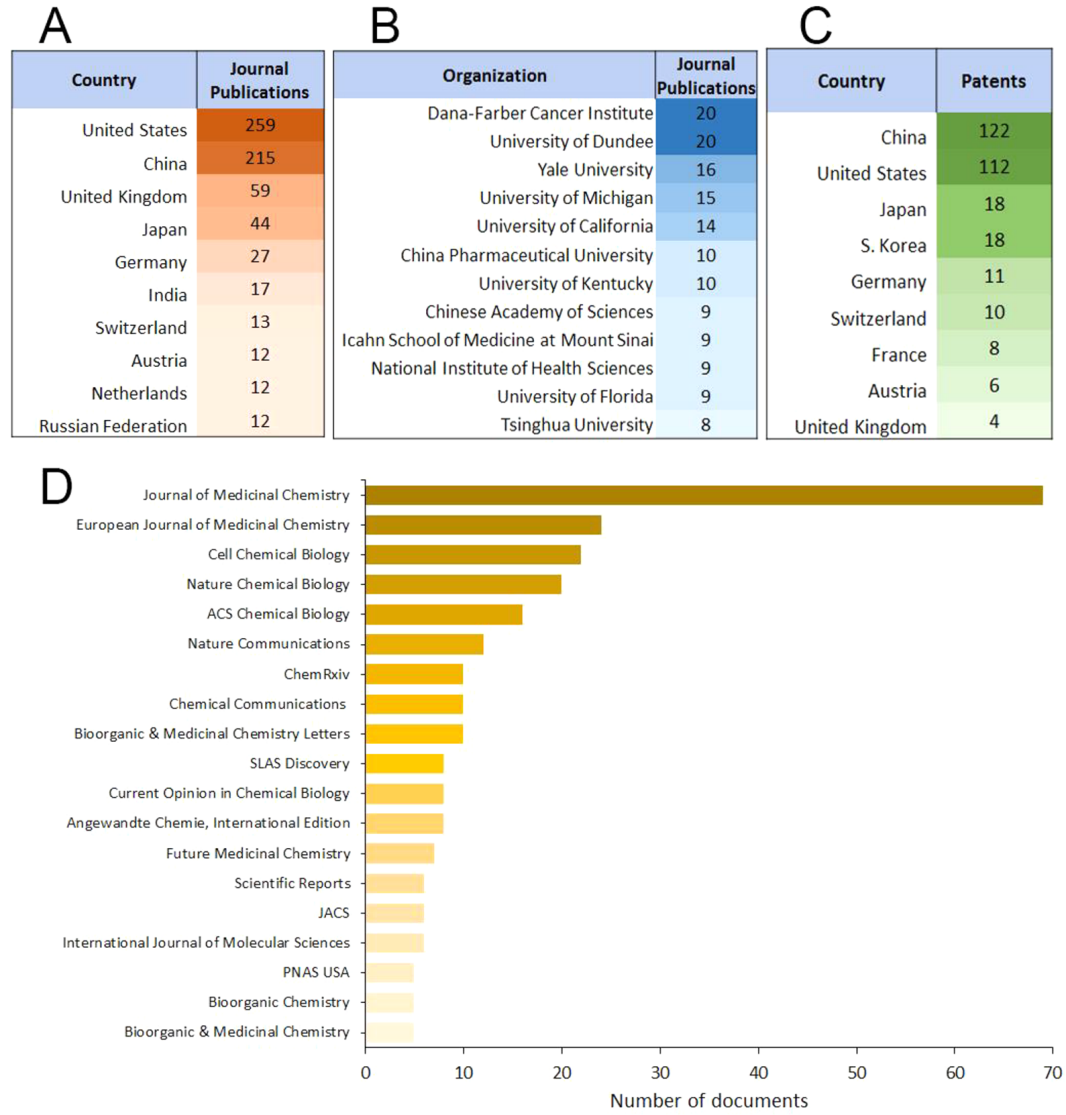
Top (A) countries, (B) organizations, and (D) scientific journals publishing TPD-related journal articles and (C) top countries filing TPD-related patents.

The largest numbers of journal publications are from authors from the United States, China, the United Kingdom, Japan, Germany, and others, as illustrated in Figure 4A. The recipients of the most protein degrader-related patent filings are China and the United States (Figure 4C), while authors from Dana-Farber Cancer Institute and the University of Dundee have published the largest number of TPD-related journal articles (Figure 4B). Figure 4D presents a list of journals that frequently publish TPD-related articles.

**Figure 5:**
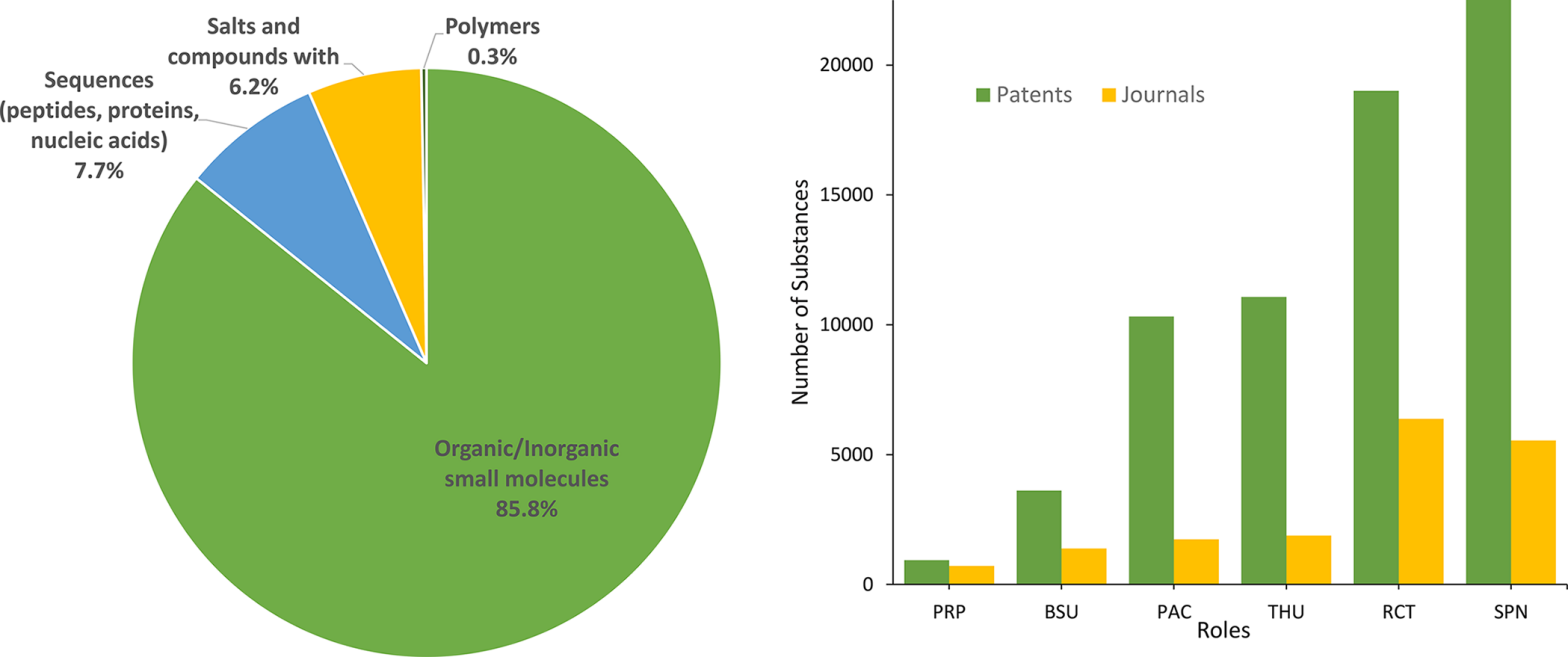
Classes of substances represented in the TPD-related documents (left) and their role indicators according to the CAS Content Collection (right) [SPN, synthetic preparation; RCT, reactant; THU, therapeutic use; PAC, pharmacological activity; BSU, biological study (unclassified); PRP, properties].

Using the CAS Content Collection data, the classes of compounds represented in the TPD-related documents and their functions as specified in the related research were analyzed. Figure 5 (left panel) illustrates the relative portion of classes of chemical compounds utilized in the TPD-related research. The area is strongly dominated by small molecules, followed by biosequences, including peptides, proteins, and nucleic acids. Indeed, the early protein-targeting chimeric molecules were peptide-based,^17,58,59^ with the first invention and design of a small molecule androgen receptor degrader using nutlin-3 to recruit MDM2 published in 2008.^19^ The roles of these substances in the protein degrader-related research as identified in the CAS Content Collection are shown in the right panel of Figure 5. Because protein degraders are synthesized via multistep chemical reactions, described in the research publications and patents, the dominance of the synthesis-related roles SPN (synthetic preparation) and RCT (reactant) is justified. The next largely presented group of roles, THU (therapeutic use) and PAC (pharmacological activity), is therapy-related, reflecting the emerging role of protein degraders in medical practice. A significantly larger number of compounds indexed in the CAS Content Collection originate from patents, which are typically used to explore and provide large libraries of relevant substances and their synthesis routes.

**Figure 6:**
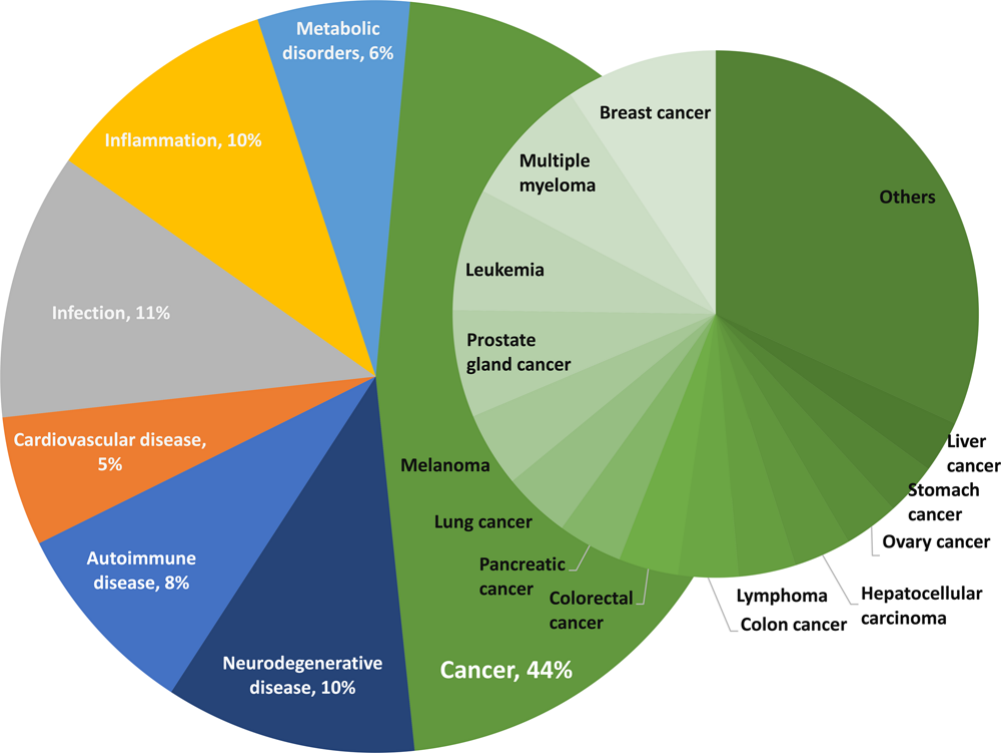
Distribution of the protein degrader-related publications in the CAS Content Collection with respect to the target diseases.

**Figure 7:**
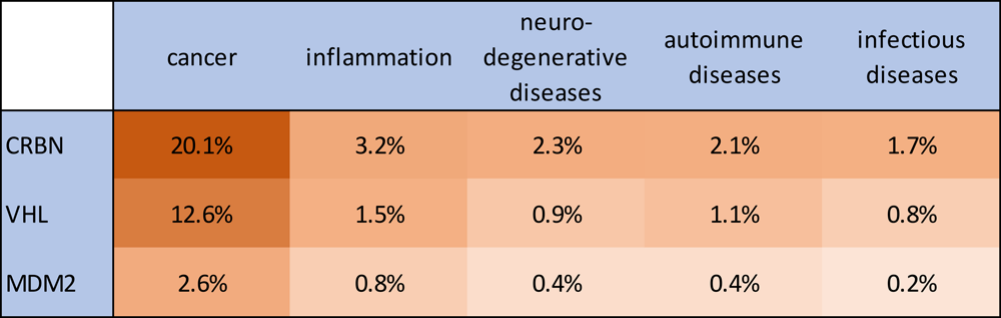
Correlation of the number of protein degrader-related publications in the CAS Content Collection for the three most widely used E3 ligases with the targeted diseases. Percentages are from the total number of protein degrader-related publications.

The motivation to explore the TPD strategy to attack diseases is the existence of a large group of undruggable disease-causing proteins that can be considered potential targets for the degraders. The variety of diseases targeted by protein degraders as revealed by our analysis of the publications in the CAS Content Collection is shown in Figure 6. The largest portion (44%) of the publications are associated with cancer treatment, with neurodegenerative, infectious, and inflammatory diseases also highly represented (Figure 6). CRBN, VHL, and MDM2 are the most popular E3 ligases being recruited by TPDs to induce ubiquitination and subsequent proteasomal degradation of a target proteins (Figure 7).

**Figure 8:**
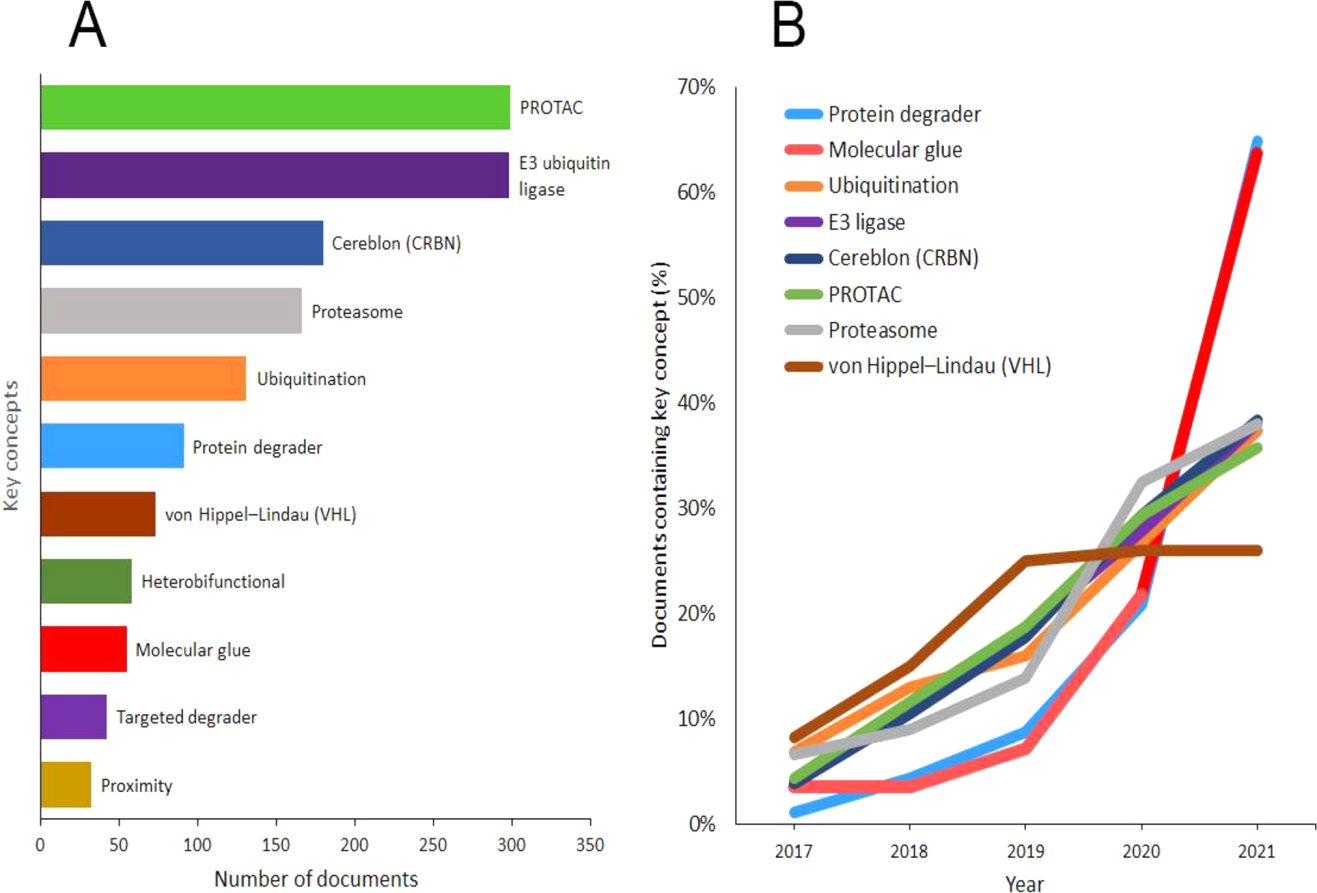
(A) Number of publications presenting key concepts related to TPDs during the years 2017–2021. (B) Trends in key concepts presented in the articles related to TPDs during the years 2017–2021. Percentages are calculated with yearly publication numbers for each key concept, normalized by the total number of publications for the same concept in the same time period.

To better reveal the rising trends in this research area, we analyzed the presence of certain key concepts in the TPD-related research in the relevant publications. Although the cumulative number of publications having “molecular glue” as a key concept is relatively small compared to the others (Figure 8A), its rate of increase in the past couple of years is significantly greater (Figure 8B), characterizing it as a trending concept in the field of small molecule drugs and their mode of action.

Thus, in view of the remarkable growth of the number of publications related to TPDs in the recent decade (Figure 3), noteworthy is one specific kind of TPD that has come to the forefront, the molecular glues, with even higher, explosive growth of interest in the past couple of years (Figure 8). In what follows, we focus on that particular highly promising kind of TPD.

## Advances in Molecular Glue Degrader Discovery

The growing research interest in molecular glue compounds is rapidly expanding the compilation of E3 ligases, molecular glues, their neosubstrates, and the associated diseases they treat, particularly for the degradation of previously undruggable proteins. Molecular glues have been discovered by serendipity, chemical library screening, and rational design. Mechanism of action, structure–activity relationship, and protein–molecular glue interaction studies of initially discovered molecular glues have laid the foundation for their optimization using structure-based drug design (SBDD).

SBDD provides a specific, efficient, and rapid process for lead compound discovery and optimization. Researchers have discovered highly potent and selective molecular glues with SBDD strategies, such as crystallization, *in silico* modeling, computational docking analysis, rationally designed chemical library constructions, biochemical screening, phenotypic screening, and structure–activity relationship analysis (Table 2).

**Table 2:** Pathway of Molecular Glue Degrader Discovery and Structure-Guided Drug Design

initial discovery	scaffold definition	optimization	validation
serendipitous^30^	crystallography^63,64^	protein–protein interaction assay of scaffold analogues^68^	binding assays^69^
high-throughput screens (HTS)^52,60,61^	molecular docking^65,66^	E3 ligase-dependent activity assay of scaffold analogues^66,67^	biochemical methods validating target degradation^46,67^
data mining^62^	structure–activity relationship (SAR) studies^66−68^		cell-based activity assays^68^
			molecular docking analysis^67^
			crystallography^69,70^

## Initial Discovery

The success of thalidomide working as a molecular glue proves the concept of E3 ligase-based targeted protein degradation as an effective therapeutic strategy, thus giving confidence to the expansion of the discovery of new molecular glues, E3 ligases, and their neosubstrate targets. Efforts to develop rational strategies for discovering new molecular glue degraders and widen the bottleneck of serendipitous discovery have led to the emergence of several successful approaches relying on high-throughput chemical library screenings. One such strategy successfully identified four new molecular glue degraders, dCeMM1–4, by screening a library of 2000 cytotoxic/cytostatic small molecules for compounds with E3-dependent antiproliferative activity.^60^ As shown in Table S1, dCeMM1 shares similar aryl sulfonamide structure with other RBM39 degraders such as Indisulam, recruiting RBM39 to the DCAF15 subunit of CRL^DCAF15^.^28^ dCeMM2–4 are cyclin K degraders that stabilize cyclin-dependent kinase 12 (CDK12)–cyclin K binding to DDB1CUL4B E3 at the CDK12–DDB1 interface. While dCeMM2 and -3 are structurally novel and similar to each other, dCeMM4 shares similarity with other cyclin K degraders such as Glue01 (Table S1). In another rational discovery approach, a library of 350 000 chemical compounds was screened using a fluorescence polarization-based binding assay, to detect substances that enhance the PPI of oncogenic transcription factor β-catenin and its E3 ligase, SCFβ-TrCP.^52^ Two lead compounds, NRX-252114 and NRX-252262 (Table S1), were designed using SBDD from four initially identified first-generation compounds with a conserved 6-trifluoromethylpyridone bound to a biaryl amide chemical scaffold.^52^ In another approach, database mining was used to screen for correlations between the cytotoxicity of a 4518-small molecule chemical library and the mRNA levels of 499 E3 ligase components against 518 human cancer cell lines. This strategy led to the discovery of compound CR8 (Table S1), which depletes cyclin K by acting as a molecular glue stabilizing the CDK12–cyclin K and DDB1 complex.^62^

Advances made using structure-based drug optimization to develop thalidomide-based analogues with reduced teratogenicity,^71^ enhanced potency, and better target specificity have led to the successful development of promising new therapeutics currently ranging from the preclinical stage to the Phase II clinical stage: CC-122,^46^ CC-220,^70^ CC-90009,^68^ CC-92480,^72^ ZXH-1-161,^67^ and SJ6986^66^ (Table S1). A structure similarity analysis of the CAS patent database for compounds with 90% similarity to thalidomide using ChemScape software within SciFinder^n^^73^ (Figure S1) shows significant numbers of recent patents related to thalidomide-based analogues. Of these 219 compounds identified, the top eight with the most frequent patent associations all retain the essential glutarimide moiety that is necessary for CRBN E3 ligase binding. Substitutions at positions C4–C6 of the phthaloyl ring and variation of the C3 carbonyl influence neosubstrate binding specificity and degradation potency. These ChemScape results highlight the research interest and relevance of thalidomide analogue drugs.

The successful discovery of these lead thalidomide analogues, also termed cereblon E3 ligase modulation drugs (CELMoDs), serves as a paradigm for using SBDD in the development of lead molecular glue degraders. Next, we will review the SBDD of CELMoDs from thalidomide, pomalidomide, and lenalidomide as an example to illustrate steps for the development of molecular glue degraders, including scaffold definition, identification of optimal scaffold analogues, and validation of lead compounds.

## Scaffold Definition

**Figure 9:**
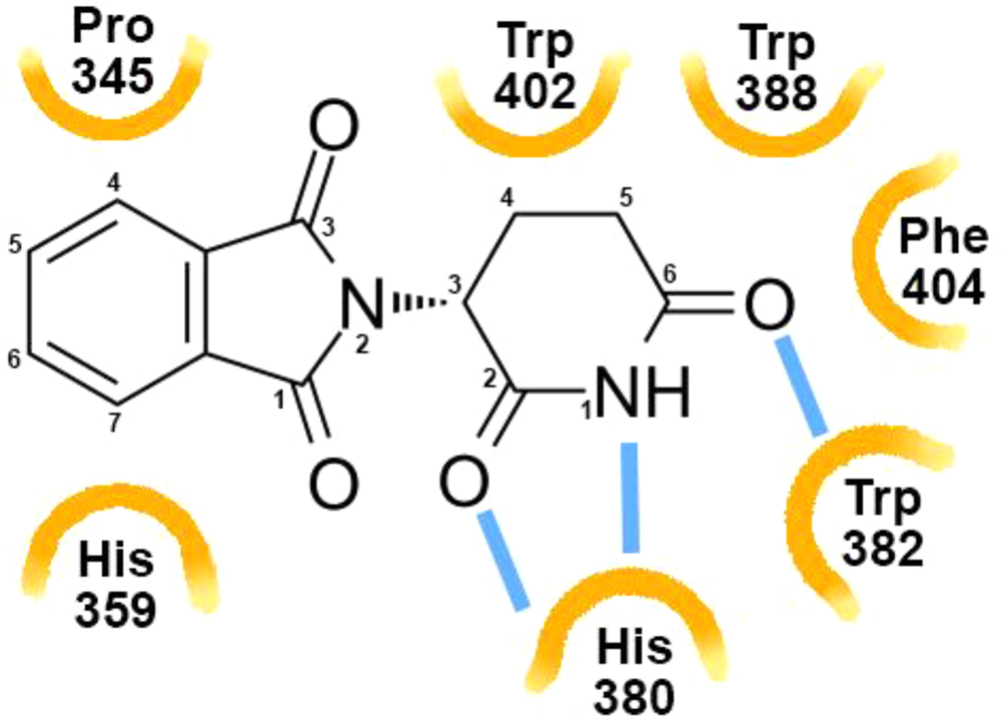
Scheme of interactions of thalidomide with CRBN (*Gallus gallus*). Hydrophobic interactions are depicted as orange semicircles, and hydrogen bonding is depicted as blue lines.

Via combination of crystallization and mutational analysis, the foundations for understanding the interaction of CRBN with thalidomide and its analogues lenalidomide and pomalidomide were laid.^26,63,64^ These studies showed how the main pharmacophore structure, the conserved glutarimide ring, binds to CRBN (Figure 9).^63,64^ It occupies a hydrophobic binding cavity between two CRBN β sheets, and the carbonyls at C2 and C6 and the amide at N1 form hydrogen bonds with CRBN. C3–C5 are in van der Waals contact with a tritryptophan hydrophobic pocket.^63^ Tyr386Ala and Trp388Ala amino acid substitution mutations altered the integrity of the CRBN binding cavity and disrupted the binding between CRBN and the glutarimide ring, eliminating interaction of all three IMiDs with CRBN and resulting in thalidomide and lenalidomide drug insensitivity *in vivo*. Positions C4–C6 on the phthaloyl ring are solvent facing, and differences in structural features at C4–C6 influence both neosubstrate specificity and potency. For example, the amide at position C4 in lenalidomide and pomalidomide specifies degradation of transcription factors IKZF1 and IKZF3 with a higher potency than thalidomide, which lacks the C4 amide, while methyl and chloro substitutions at position C4 increase the rate of degradation of IKZF1. Substitutions at C5 or C4 and C6 diminish IKZF1 degradation.^63^ This work has been serving as the foundation for molecular glue-related SBDD.

## Optimization of Specificity and Potency

Efforts to further explore the influence of phthaloyl ring modifications on neosubstrate binding, degradation, and antiproliferative activity have led several groups to focus on identifying thalidomide analogues with enhanced potency and specificity. Bristol Myers Squibb (BMS)/Celgene’s second-generation compounds CC-122, CC-220, and CC-885 were synthesized as part of various focused combinatorial libraries retaining the main CRBN binding pharmacophore glutarimide ring with variations of the solvent-exposed ring. CC-122 (Table S1) was synthesized as part of their quinazolinone–glutarimide derivative library.^74^ Compared to lenalidomide, CC-122 shows enhanced proteasome-dependent degradation of IKZF1 and IKZF3 with broader and enhanced antiproliferative activity across both ABC- and GCB- DLBCL cell lines.^46^ CC-220 (Table S1) was synthesized as part of BMS/Celgene’s 4′-arylmethoxy isoindoline–glutarimide library.^75^ Studies show CC-220 binds to CRBN with a higher affinity than lenalidomide and pomalidomide in time-resolved fluorescence energy transfer cereblon binding assays and degrades IKZF1 and IKZF3 with greater potency in cell-based chemiluminesence substrate degradation assays.^70^ CC-885 (Table S1), synthesized as part of BMS/Celgene’s 5-substituted isoindoline–glutarimide library,^76^ was identified with strong CRBN-dependent antiproliferative activity in a broad range of tumor cell lines, with enhanced antiproliferative activity compared to that of lenalidomide and pomalidomide in 12 AML cell lines studied. Immunoprecipitation assays and immunoblotting assays show that CC-885 promotes the binding of CRBN to a novel substrate, GSPT1, targeting its degradation.^69^

Concerns about the poor toxicity profile of CC-885 led BMS/Celgene to further explore the development of analogues of CC-885 with better antiproliferative activity against a broad panel of AML cell lines. Using CC-885 as a structural template, SAR studies defined a scaffold with a difluoro acetamide linker that maintained a good *in vitro* selectivity index. From this scaffold, a focused library of a series of difluoro acetamide analogues was synthesized and screened, leading to the identification of BMS/Celgene’s third-generation lead compound, CC-90009.^68^ Validation studies further demonstrated that CC-90009 shows strong potency and specificity in degrading GSPT1, not IKZF1 or IKZF3. A similar approach was used to explore rationally designed molecular glue degraders showing specificity for IKZF1 and -3 with higher potency and more rapid degradation profiles than lenalidomide in the treatment of relapsed or refractory multiple myeloma (RRMM). These studies led to the successful identification of an additional third-generation molecular glue degrader, CC-92480 (Table S1).^72^ When the CRBN modulator library was screened for selective antiproliferative activity in a lenalidomide-resistant MM cell line, lead compound 13 was identified and its chemical structure was used as the foundation for SAR studies, distinguishing the minimal pharmacophore necessary to maintain strong antiproliferative activity and rapid IKZF3 degradation. The defined scaffold retains the essential features of the glutarimide ring for binding CRBN, an isoindoline ring similar to lenalidomide with a 4-oxy substitution in place of the 4-amine, and *S* chirality, while allowing variability on the terminal arylpiperazine. The inspection of a series of arylpiperazine analogues identified CC-92480 as a lead compound for clinical development with less off-target binding compared to compound 13, specific IKZF1 and IKZF3 degradation, and more potent and rapid degradation profiles compared to those of lenalidomide or pomalidomide.^72^

Further, additional CELMoDs were identified by screening a focused combinatorial library of 51 compounds with three lenalidomide heterocyclic scaffolds for CRBN-dependent antiproliferative activity in a MM cell line.^67^ Expression proteomic validation studies of compounds of interest confirmed target specificity. Lead compound ZXH-1-161 was identified, with antiproliferative activity in a MM cell line and improved selectivity for GSPT1 degradation compared to that of CC-885.^67^ Nishiguchi et al. recently explored the rational design of novel CRBN-dependent molecular glue degraders by screening a chemical library constructed using the essential thalidomide pharmacophore defined from crystallization and SAR studies of IMiDs.^66^ A 415-compound focused chemical library was synthesized from 30 thalidomide analogue scaffolds, and both the landscape of the library by *in silico* molecular docking analysis and the physiochemical descriptors of included analogues were evaluated prior to phenotypic screening. Lead CELMoD compound SJ6986 was identified with antiproliferative activity against a broad range of cell lines, potent degradation of GSPT1 and -2 with selectivity over IKZF1 and -3, and high bioavailablility in mice. This success validates the application of chemical docking strategies to confirm spatial diversity within the binding cavity and calculation of physicochemical descriptors to ensure analogues lie within a drug-like property space, prior to focused combinatorial library synthesis and screening for activity.^66^

## Validation

Once favorable drug candidates are identified, their therapeutic specificity and potency need to be further validated. The modes of action of these leading drug candidate compounds are confirmed, and further functional bioactivity testing is performed. Chemical interactions, degradation target specificity, CRBN-dependent antiproliferative and/or other therapeutic activity, cell line specificity, and pharmacokinetic/pharmacodynamic profiles are validated using a variety of biochemical, genetic, pharmacological, or degradation assays. Validation assays to confirm the chemical interactions of lead CELMods have included crystallography,^69^ docking analysis,^66^ fluorescence polarization assays,^66^ TR-FRET,^67^ and co-precipitation^69^ or pull-down assays.^69^ Targets of degradation have been validated by immunoblotting,^66−68^ chemiluminescence-based assays,^70^ and expression proteomic analysis.^66,67^

Confirmation of the cellular activity of CELMods of interest has included antiproliferation assays across specific cell lines to validate the cell line specificity, potency, and therapeutic value of lead compounds.^66,68^

## Identification of New Targets for Thalidomide Analogue Molecular Glue Degraders

E3 ubiquitin ligases recognize their substrates through degrons, short sections of primary protein sequence that are necessary and sufficient for the interaction with substrate receptors of ubiquitin ligases.^77^ Chemoproteomics provide a useful approach for identifying proteins from the human proteome with favorable degron features, making these targets feasible candidates for recruitment to CRBN. IKZF1 and IKZF3 zinc finger proteins are essential transcription factors in multiple myeloma. After the identification of a single Cys2-His2 (C2H2) zinc finger fold as the minimal sufficient degron necessary for IKZF1/IKZF3 degradation, the entire human C2H2 zinc finger proteome (>800 proteins) was screened for targets for thalidomide analogue-mediated degradation. Six proteins recruited for degradation were identified (including IKZF1/IKZF3), and four of them were newly identified CRBN targets for thalidomide analogues.^78^

In addition, through computational *in silico* docking analysis of all human C2H2 zinc fingers with CRBN, using the pomalidomide–CRBN crystal structure for structural similarity, approximately 50–150 CRBN binding zinc finger candidates were identified; 33 of these zinc fingers were further tested for *in vitro* CRBN–pomalidomide binding, and 28 (a remarkable 85%) tested positive for binding. New targets of therapeutic interest can be examined further to identify the optimal thalidomide analogue molecular glue degraders that stabilize the PPIs of CRBN with neosubstrates of interest. Sources of these optimal degraders could potentially be searched for among existing focused chemical library collections or through constructing new *in silico* assisted custom-designed chemical libraries tailored for chemical feasibility and spatial diversity within the new CRBN–neosubstrate binding cavity space with physicochemical descriptors that lie within the drug-like property space.^78^

A chemical structure similarity search based on the thalidomide analogue CC-885, performed by SciFinder-n,^73^ found 310 compounds within the 85–99% similarity range, exhibiting a wide variety of bioactivities, including antitumor, neurological, anti-infective, cardiovascular, etc. (Figure S2). Additionally, substructure searches can be useful for exploring previously synthesized analogues for inclusion in the construction of desired focused chemical libraries. A substructure search using an essential α,α-difluorobenzeneacetamide CC-885 analogue scaffold identified 715 compounds retaining this exact chemical scaffold (Figure S3). SciFinder-n offers the possibility of screening the prospective library compounds for Lipinski properties and structure-related properties such as the lipophilicity descriptor (log *P*), molecular weight (MW), polar surface area (PSA), H-bond donors (HBD), and H-bond acceptors (HBA), confirming that physicochemical descriptors lie within a drug-like chemical property space, prior to library construction. Commercial availability and reaction synthesis details can also be explored.^73^ Thus, SciFinder-n appears to be a powerful resource for the design of focused chemical libraries of potential molecular glues mediating the PPI of E3 ligase subunits and new prospective targets.

## Discovered Molecular Glues, E3 Ligases, and Target Proteins

Small molecules that bind the E3 ligase CRBN are the most investigated molecular glues. In addition to them, there are other molecular glues that induce protein degradation through various mechanisms of action, including autophagy-mediated protein degradation. A selection of promising E3 ligase molecular glues, non-E3 ligase molecular glues, and natural molecular glues are examined. More detailed information about these molecular glues is listed in Table S1.

## E3 Ligase Utilizing Targeted Protein Degraders

### Degradation of Transcription Factors IKZF1 and IKZF3

IKZF1 and IKZF3 are lymphocyte lineage transcription factors^79,80^ that are key regulators for the survival of the malignant plasma cells in multiple myeloma. IKZF1 and IKZF3 are considered as undruggable target proteins due to the lack of druggable binding pockets. Acting as molecular glue, thalidomide and its analogues, lenalidomide (Revlimid) and pomalidomide (Pomalyst), induce the formation of a CUL4–DDB1–RBX1–CRBN E3 ligase complex. This ternary complex promotes ubiquitination and degradation of IKZF1 and IKZF3. Degradation of IKZF1 and IKZF3 causes inhibition of the proliferation of multiple myeloma cells and suppression of the differentiation of B-cells. These three agents are approved by the U.S. FDA to treat multiple myeloma and del(5q) MDS.^64,81−84^ Molecular glue compounds CC-122, CC-220 (iberdomide), and CC-99282 (Table 3) degrade IKZF1 and IKZF3 through binding to CRBN E3 ligase.^46^ These compounds are currently in Phase I/II clinical trials for treatment of multiple myeloma, non-Hodgkin’s lymphoma, and systemic lupus erythematosus.^70,85−87^ CFT7455 (Table 3) is a next-generation IKZF1 and IKZF3 degrader that binds to CRBN E3 ligase. CFT7455 exhibits favorable physiochemical properties, pharmacokinetic parameters, and good oral bioavailability in preclinical studies.^14^ CFT7455 is more potent and catalytically active than other approved IMiDs and is currently in multiple clinical trials for treatment of multiple myeloma and relapsed/refractory non-Hodgkin’s lymphoma.^88^ DKY709 (Table 3) as a CRBN binder induces the formation of the CRBN–DKY709–IKZF2 ternary complex. This ternary complex promotes ubiquitination and degradation of IKZF2. DKY709 in combination with spartalizumab is currently in clinical trial in patients with advanced solid tumors, including melanoma.^89^

**Table 3:**
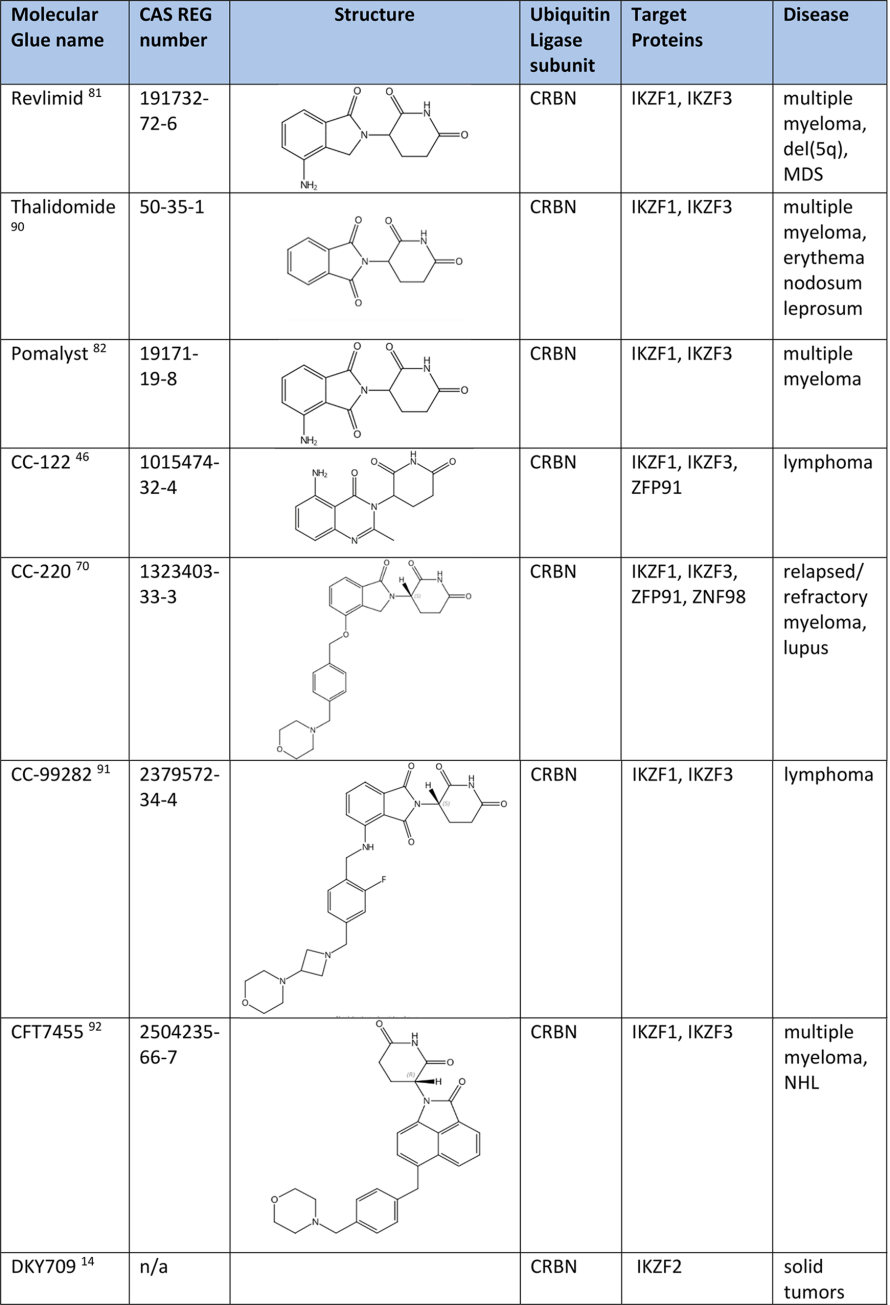
Degraders of Transcription Factors IKZF1 and IKZF3

### Degradation of Cyclin K and CDK12

Cyclin K and CDK12 are promising drug targets for treatment of cyclin E1-overexpressing tumors of human tumorigenesis.^93^ Molecular glues trigger the polyubiquitination and subsequent degradation of CDK12 ’s partner protein CCNK through binding to CDK12 and recruiting CCNK to form ternary complexes.^61^ Several small molecules have been explored as molecular glues that modulate the binding of CDK12 protein to DDB1 in the DDB1–CUL4–RBX1 complex. For example, CR8, gluing DDB1 to cyclin K, induces cancer cell apoptosis and has neuroprotective effects (Table 4).^62,94,95^ The pyridine moiety in the CDK-bound form of CR8 induces the formation of a complex between CDK12–cyclin K and the CUL4 adaptor protein DDB1, resulting in ubiquitination and degradation of cyclin K. Other promising cyclin K degraders include a series of 5-methylthiazol analogues [glue01 series (Table 4)],^96,97^ dCeMM compounds (dCeMM2–4),^61^ and HQ005. HQ005 is a leading molecular glue drug candidate discovered by structure optimization of HQ461 (Table S1). It glues DDB1 to CDK12 for cyclin K degradation (Table 4).^26,61^

**Table 4:**
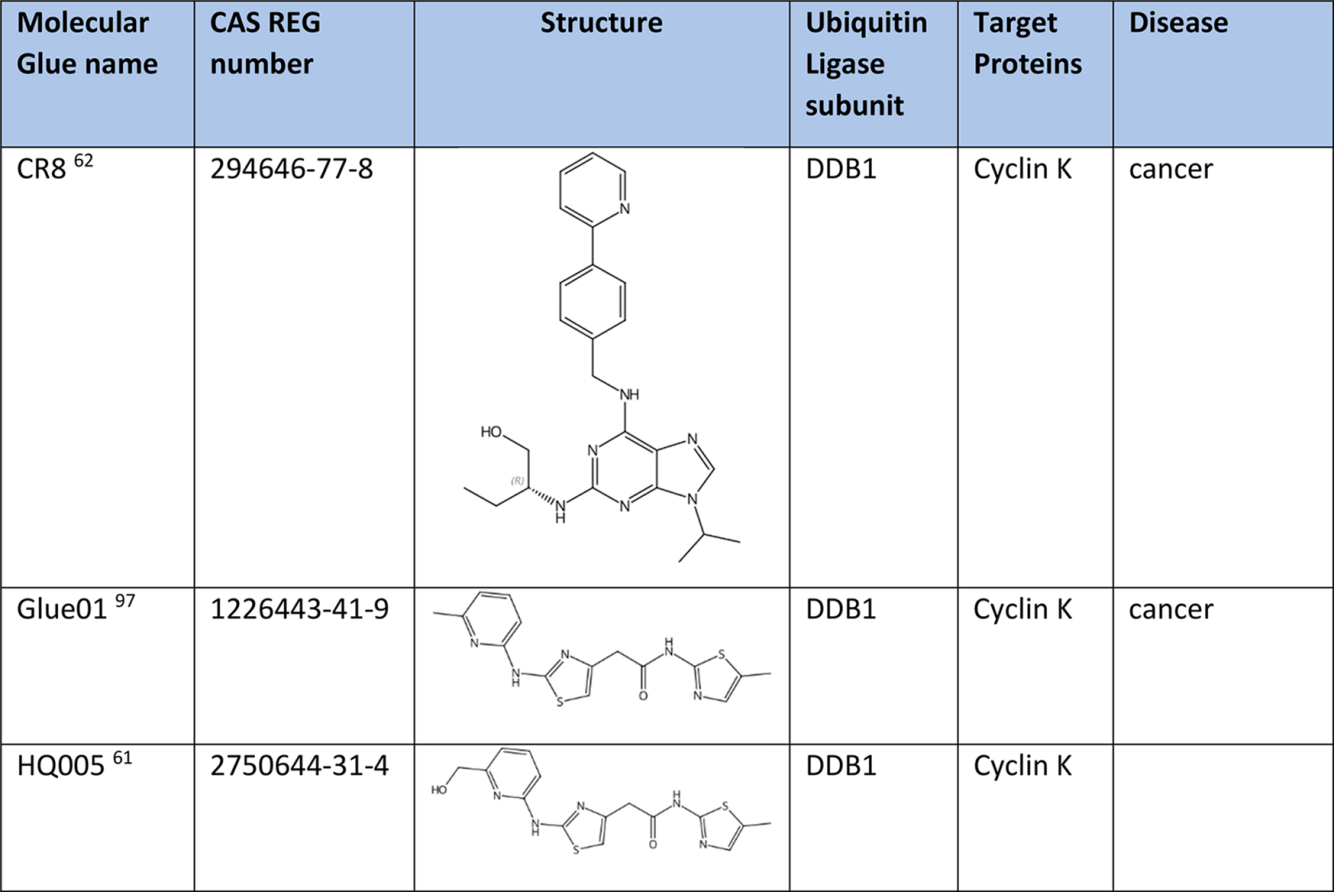
Degraders of Cyclin K and CDK

### Degradation of Casein Kinase 1α (CK1α)

CK1α (encoded by *CSNK1A1* in humans) is a member of the CK1 family of proteins. It regulates various signaling pathways involving autoimmune diseases, neurodegenerative diseases, and cancer. FPFT-2216 and TMX4116 (Table 5)^96^ each induce the formation of ternary complexes involving CRBN E3 ligase and CK1α. The formation of this ternary complex promotes ubiquitination and degradation of CK1α. FPFT-2216 is a nonselective CK1α degrader. In addition to CK1α, it degrades IKZF1, IKZF3, and PDE6D. In contrast, TMX-4116 is a specific CK1α degrader, although it was discovered from structural modification of FPFT-2116. Both agents act as molecular glue CK1α degraders and are being used in therapeutic applications for treatment of multiple myeloma.

**Table 5:**
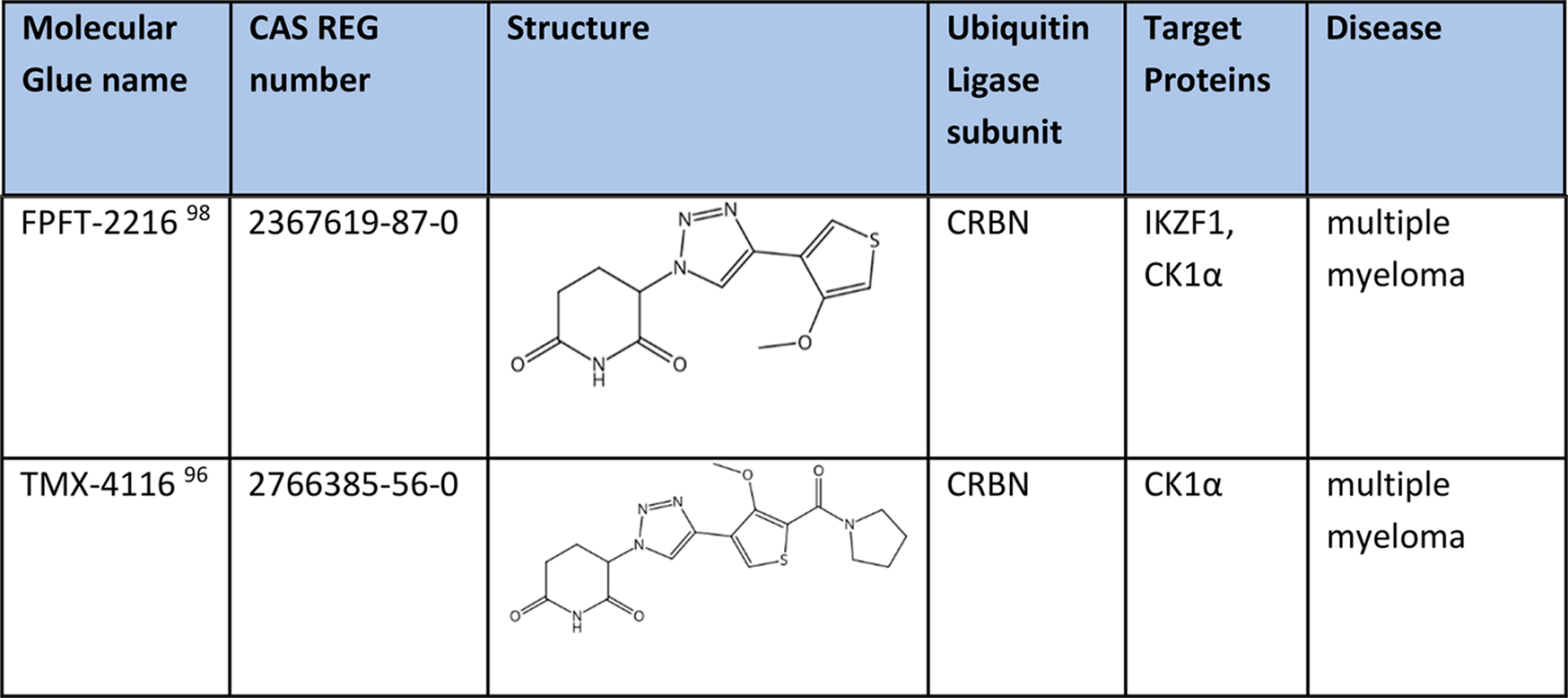
Degraders of CK1α

### Degradation of G1 to S Phase Transition Protein 1 (GSPT1)

Translation termination factor GSPT1 is overexpressed and oncogenic in several cancers.^99^ GSPT1 is currently being explored as a therapeutic target for the treatment of acute myeloid leukemia. Molecular glues such as certain new CRBN modulators have shown the ability to induce selective degradation of GSPT1. CC-90009 [eragidomide (Table 6)], structurally optimized from CC-885,^69^ is the first rationally designed clinical candidate driven by the molecular glue-degrading mechanism.^68^ Currently, CC-90009 is the first CRBN-mediated protein degrader in Phase I clinical trials for treatment of relapsed/refractory acute myeloid leukemia.^14^ The sulfonamides SJ6986 and SJ7023, discovered by cell-based phenotypic screening of a library, have shown antiproliferative activities in two leukemia cell lines. Both compounds were identified as CRBN binders to recruit GSPT protein, forming ternary complexes and further inducing GSPT1 and -2 degradation.^66^ BTX-1188 and MG-277 (Table 6) induce the degradation of GSPT1 to achieve their potent anticancer activity. MG-277 is a powerful antitumor agent suppressing tumor cell growth in a p53-independent manner.^33^ BTX-1188 is a leading molecular glue drug candidate in Phase I clinical trials for treating acute myeloid leukemia and myelodysplastic syndrome. It is worth pointing out that BTX-1188 is discovered to degrade GSPT1, IKZF, and CK1α and is expected to kill tumor cells and simultaneously modulate the immune system, resulting in better efficacy and potentially fewer side effects.^100^ ZXH-161 (Table 6) is a leading drug candidate exhibiting better potency and selectivity in GSPT1 degradation. Current research on ZHX-161 is exploring the opportunities for therapeutically targeting GSPT1, considering that GSPT1 degradation has already shown significant potential in the treatment of acute myeloid leukemia.^101^

**Table 6:**
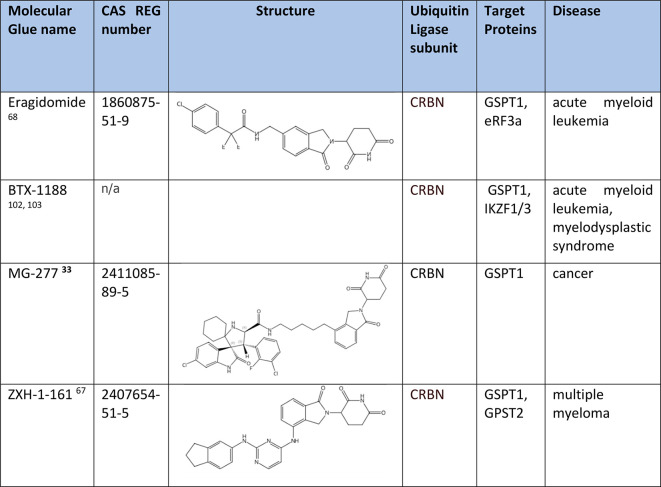
Degraders of GSPT1

### Degradation of Sal-like Protein 4 (SALL4)

SALL4, a *spalt*-like developmental transcription factor, is important for limb development.^104^ Thalidomide and its derivatives induce degradation of SALL4, which is the likely reason for the observed birth defects detected upon the initial introduction of the drug. These findings can inform the development of new compounds that induce CRBN-dependent degradation of disease-relevant proteins but avoid degradation of developmental transcription factors such as SALL4 and thus have the potential for therapeutic efficacy without the risk of teratogenicity, a defining feature of this class of drugs.^105^

### Degradation of RNA Binding Motif Protein 39 (RBM39)

RBM39 is an RNA binding protein involved in transcriptional co-regulation and alternative RNA splicing. Recent studies have revealed that RBM39 is the unexpected target of aryl sulfonamides, indisulam, E7820, and CQS (Table 7), which act as molecular glues between RBM39 and the DCAF15-associated E3 ubiquitin ligase complex leading to selective degradation of RBM39. Loss of RBM39 leads to aberrant splicing events and differential gene expression, thereby inhibiting cell cycle progression and causing tumor regression in a number of preclinical models.^106^ Indisulam, E7820, and CQS have been evaluated in clinical trials as antitumor drug candidates.^47,107^

**Table 7:**
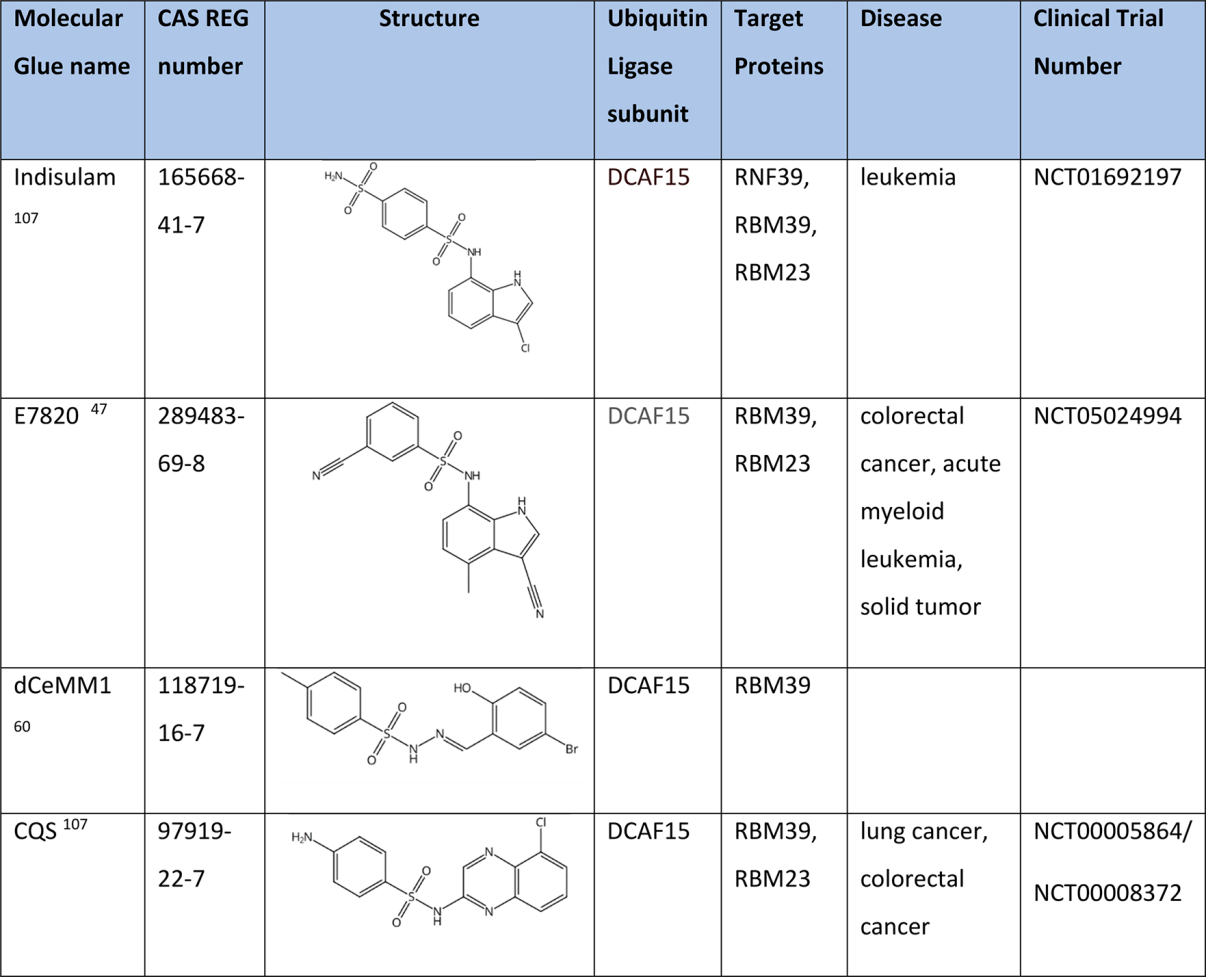
Degraders of RBM39

### Degradation of β-Catenin

Oncogenic transcription factors remain extremely challenging proteins to target, despite being implicated in multiple diseases. β-Catenin is the Wnt signaling effector protein that is often dysregulated and stabilized in cancer.^108,109^ NRX-252114 and NRX-252262 (Table 8) are leading compounds discovered recently with suitable druggabilities and enhanced binding affinity for pSer33/S37A β-catenin peptide for β-TrCP. The enhanced PPI affinity results in enhanced K48-linked ubiquitylation of mutant β-catenin by its natural ubiquitin ligase SCF^β-TrCP^, thereby promoting its proteasomal degradation.^52^

**Table 8:**
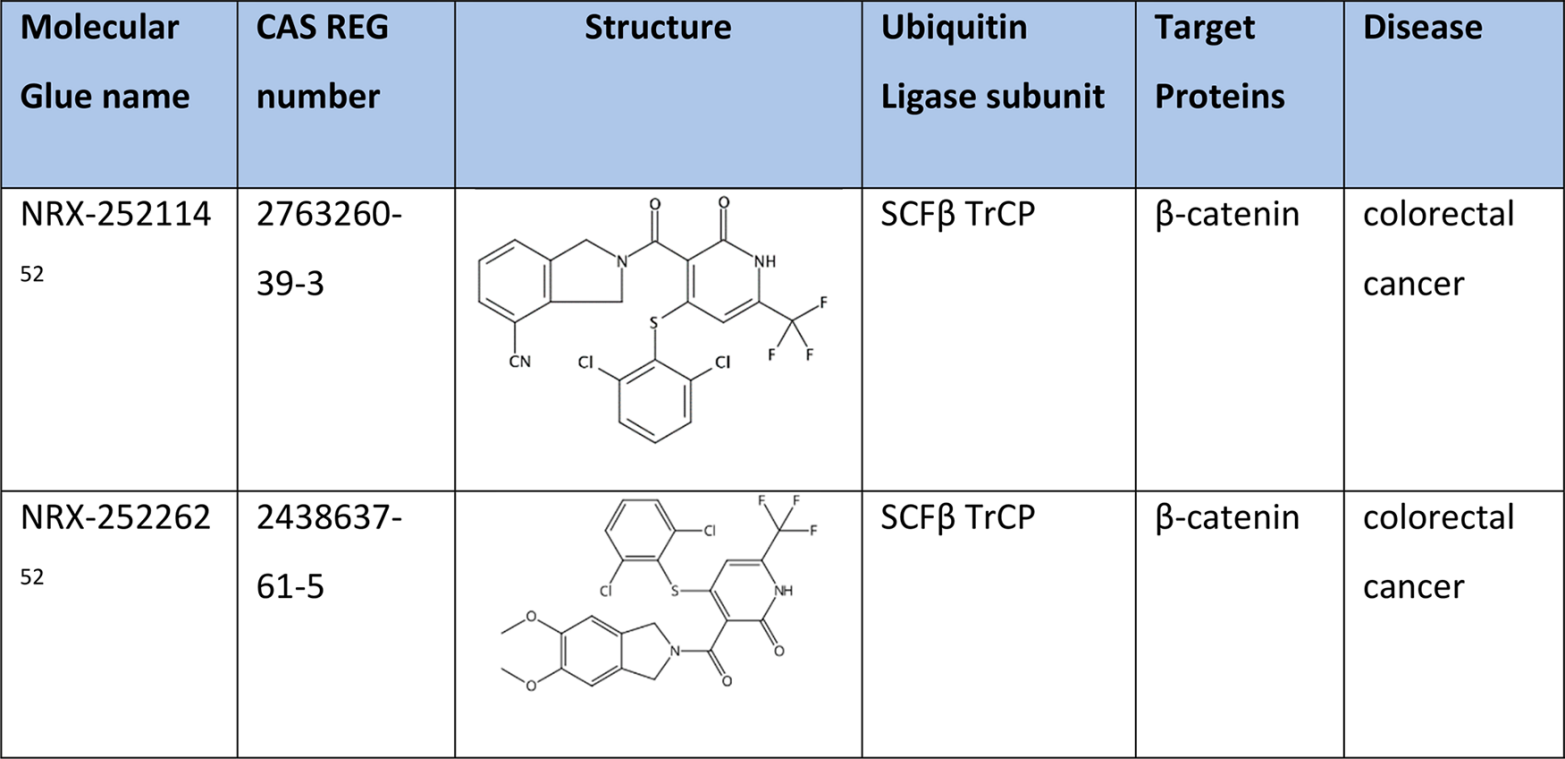
Degraders of β-Catenin

### Degradation of BCL6 Protein

Targeting BCL6 protein is an effective therapeutic approach for treating diffuse large B-cell lymphoma (DLBCL).^110,111^ BI-3802 (Table 9) induces polymerization of BCL6 and interaction between BCL6 and SIAH1 E3 ligase. SIAH1 mediates ubiquitination and degradation of polymerized BCL6.^112,113^ SIAH1 recognizes the VxP motif and exhibits weak affinity for BCL6. The binding affinity between polymerized BCL6 and SIAH1 is significantly enhanced. CCT369260 (Table 9), an analogue of BI-3802 with improved physiochemical properties, has progressed into pharmacokinetic studies. The results show degradation of tumoral BCL6 *in vivo* following oral dosing in a lymphoma xenograft mouse model.^114^

**Table 9:**
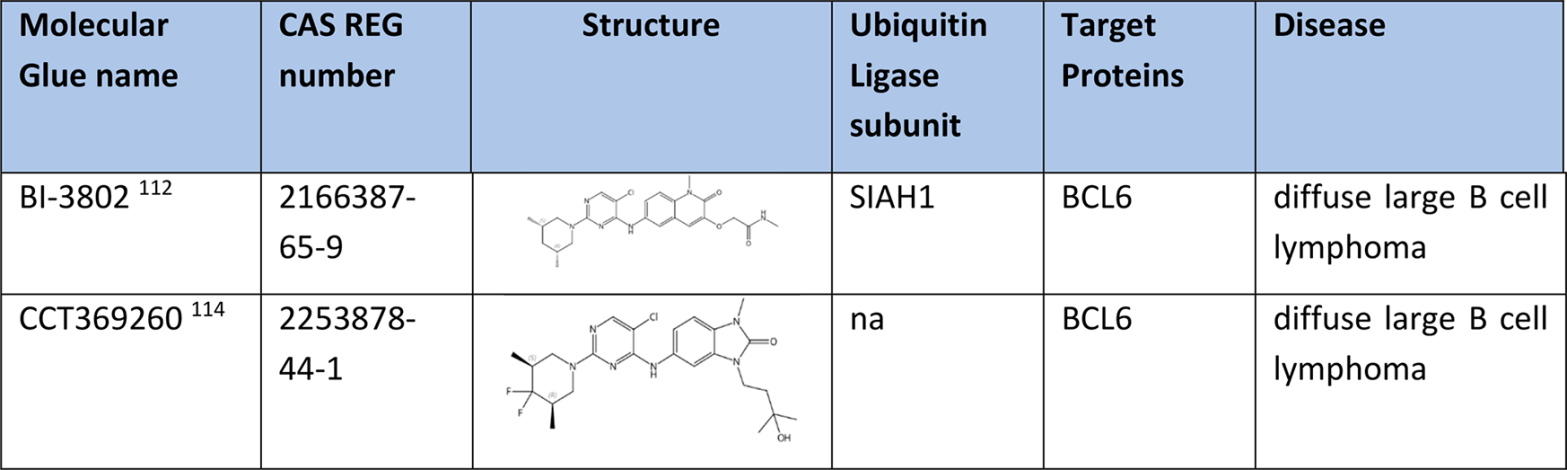
Degraders of B-Cell Lymphoma 6 Protein

**Figure 10:**
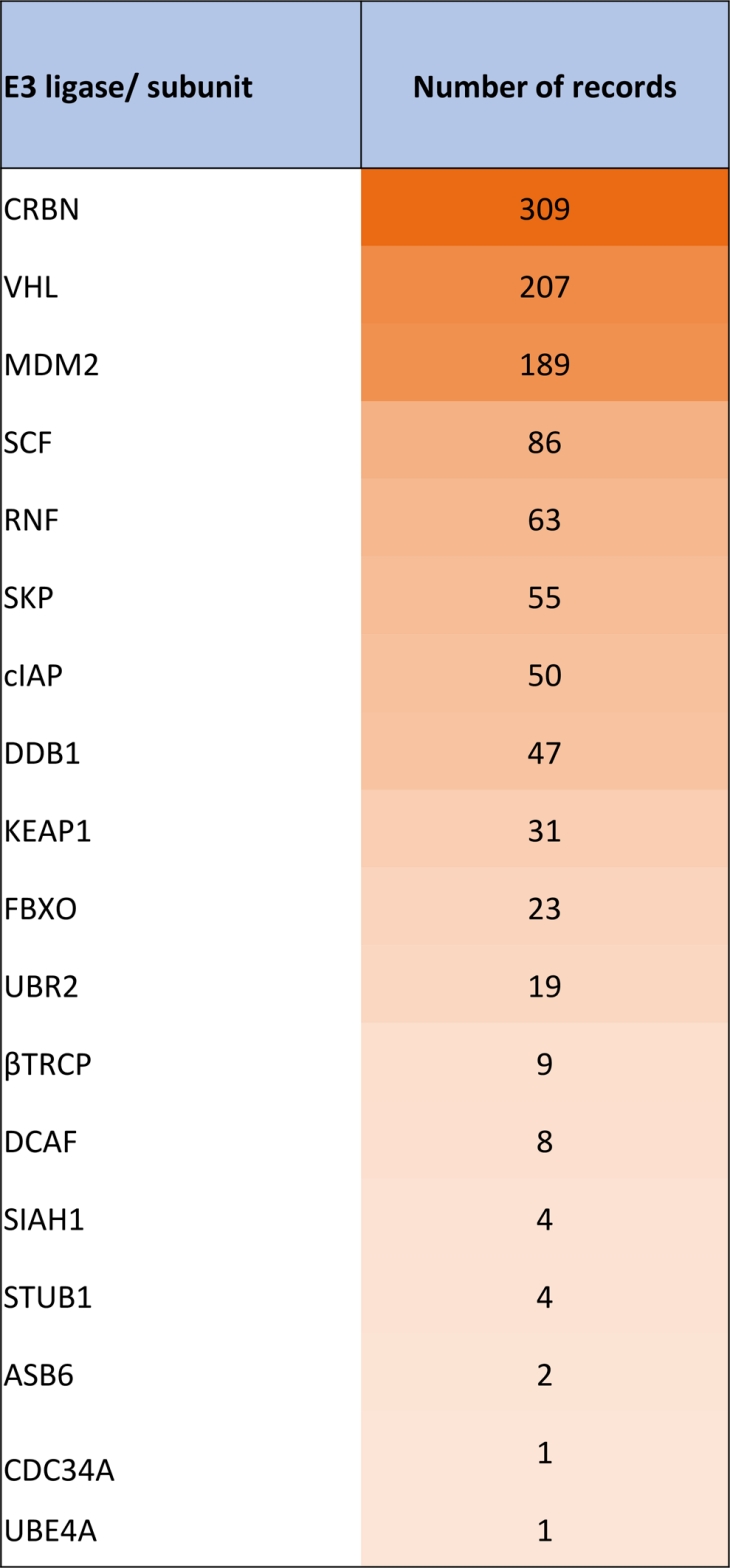
Number of documents in the CAS Content Collection related to E3 ligase recruiters exploited for targeted protein degradation.

Figure 10 summarizes the major E3 ligase recruiting TPDs as reflected in the number of documents in the CAS Content Collection.

### Degraders of Natural Molecular Glues

While molecular glues are mostly designed and synthesized in the lab, there are also some natural compounds that were found to function as molecular glues. Cyclosporin A [CsA (Table 10)] is the binding partner of the cyclophilin 18 (Cyp18)–CsA complex. The Cyp18–CsA complex recruits calcium/calmodulin-dependent serine-threonine protein phosphatase calcineurin (CN), resulting in blockage of the transcription of cytokine genes in activated T-cells. CsA is a highly specific inhibitor of T-cell activation.^115^ Voclosporin [Lupkynis (Table 10)] was approved by the U.S. FDA to treat adults with lupus nephritis. Voclosporin is a novel immunomodulatory drug inhibiting the calcineurin enzyme with the same mechanism of action as CsA.^116^ Sanglifehrin A [SfA (Table 10)] exhibits antiproliferative and immunosuppressive activity by inhibiting both T-cell and B-cell proliferation.^117^ SfA is a binding partner for the SfA–Cyp18 complex and inosine-5′-monophosphate dehydrogenase 2 (IMPDH2). Formation of the ternary complex modulates cell growth through interaction with the cystathionine-β-synthase (CBS) domain of IMPDH2.^118^ Plant hormones auxin [AUX, indole-3-acetic acid, IAA (Table 10)] and jasmonate [JA (Table 10)] are simply structured natural molecular glues. Auxin binds directly to the Skp1-cullin 1-F box (SCF) E3 ubiquitin protein ligase TIR1 and attracts AUX proteins for degradation.^119^ Jasmonate also utilizes the E3 ubiquitin ligase SCF^TIR1^ to attract and degrade jasmonate–ZIM (JAZ) domain proteins. AUX/IAA and JAZ represent families of transcriptional repressors, which upon degradation lead to the expression of auxin- and JA-inducible genes.^28,120^

**Table 10:**
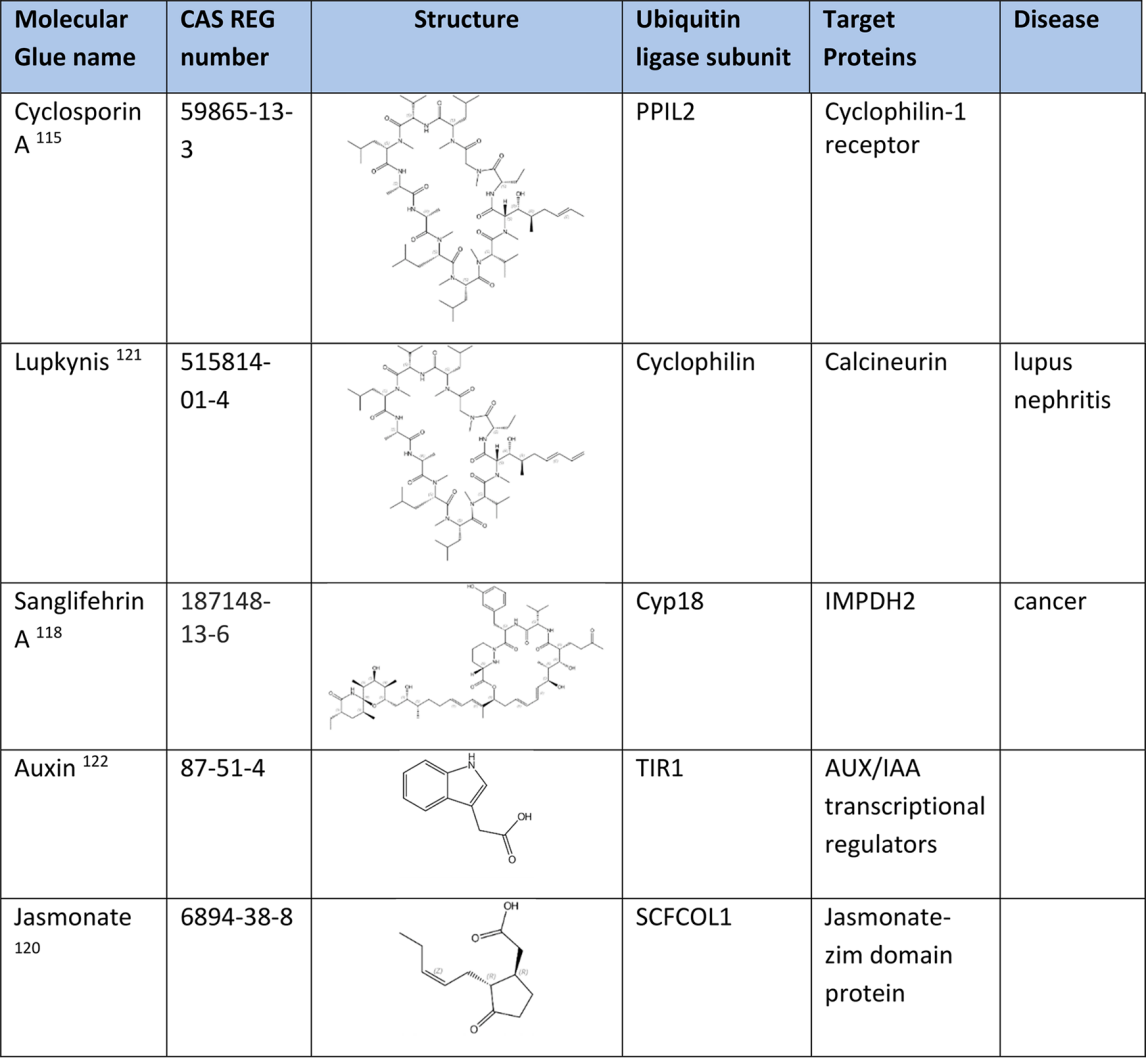
Degraders of Natural Molecular Glues

## Companies and Research Organizations Developing Molecular Glues and the Diseases They Treat

While many potential molecular glue compounds may come from drug discovery methods, few have progressed to the clinic for examination of their disease treating efficacy (Table S1). Regulatory agency-approved molecular glue treatments that have progressed to the market are even fewer. A snapshot of promising companies and research organizations is examined and highlighted to show the top players in the molecular glue drug discovery pipeline.

Companies and research organizations with a focus on molecular glue drug discovery are creating pipelines of therapeutics that are progressing to the clinic (Table 11). One of these companies, Ronok, has a promising drug candidate, RNK0507. Its investigational new drug application was cleared by the U.S. FDA in January 2022. Phase I/II studies will begin enrollment in the first half of 2022 for the treatment of advanced solid tumors and lymphomas.^123^

**Table 11:** Preclinical Molecular Glue Companies

organization	highlights
Ranok (Hangzhou, China)	drug candidate RNK05047 entering clinical trials in the first half of 2022 for treatment of solid tumors and lymphomas^123^
Monte Rosa Therapeutics (Boston, MA)	initiated IND-enabling activities for its lead program targeting GSPT1 for oncology treatment and beyond; IND application to be submitted to the FDA mid-2022; drug discovery phase for other molecular glues targeting solid/liquid tumors, autoimmune diseases, and blood diseases^128^
Plexium/partnered with Amgen (San Diego, CA)	lead optimization phase for a cereblon molecular glue targeting IKZF2 for the treatment of immune disease and cancer; drug discovery phase for a disclosed novel E3 ligase molecular glue and also undisclosed partnered molecular glue programs^129^
Frontier Medicines/partnered with AbbVie (San Francisco, CA)	drug discovery phase to develop small molecule covalent drugs against intractable immunology and oncology targets^130^
f5 Therapeutics (San Diego, CA)	pipeline of molecular candidates for hepatocellular carcinoma, breast cancer, lung cancer, head and neck cancer, colorectal cancer, gastric cancer, multiple sclerosis, rheumatoid arthritis, nonalcoholic steatohepatitis, and liver fibrosis^131^
Ambagon Therapeutics/partnered with BMS and Merck (San Carlos, CA)	drug discovery phase with five early discovery oncology treatment compounds; focusing on targeting gene signaling and expression and disrupting the cell cycle, along with other cancer-causing dysregulations, Ambagon expects to have at least one development candidate by the second quarter of 2023^132^
Captor (Wrocław, Poland)	drug candidates for hepatocellular carcinoma and autoimmune liquid tumors^133^
Amphista Therapeutics (London, U.K.)	aims to move beyond use of ubiquitin E3 ligase cereblon; they will initially focus on cancer treatments, with the possibility of branching out to treat neurological, neurodegenerative, and immunological disease along with other areas of high unmet medical need in the future^134^
Dunad Therapeutics (Cambridge, U.K.)	drug discovery phase utilizing central nervous system accessible therapeutics^135^
Proxygen/partnered with Boehringer Ingelheim (Vienna, Austria)	drug discovery phase treating lung and gastrointestinal cancers^136^
Neomorph/partnered with Dana-Farber Cancer Institute (San Diego, CA)	drug discovery phase to advance their molecular glue development pipeline against undruggable targets^137^
Seed Therapeutics/partnered with Lilly (New York, NY)	drug discovery phase with molecular glue pipeline candidates treating cancers, neurodegenerative diseases, and infectious diseases; their lead compound targets the KRAS oncogene^138^
Pin Therapeutics (Seoul, South Korea)	drug discovery phase^139^
Venquis Therapeutics, (San Diego, CA)	drug discovery phase for cancer and degenerative diseases^140^
IRB Barcelona/partnered with Almirall (Barcelona, Spain)	drug discovery phase for skin disease treatment^141^
Shanghai Dage Biomedical Technology Co., Ltd. (Shanghai, China)	pipeline of molecular glues addressing targets for cancers, inflammatory disease, and metabolic disease; lead optimization phase for oncology molecular glue candidates^142^
Triana Biomedicines (Waltham, MA)	launched recently in April 2022 to establish a rationally designed molecular glue pipeline to treat inadequately addressed diseases^143^
Evotec/partnered with BMS (Hamburg, Germany)	drug discovery phase to develop a pipeline of molecular glue degraders^144^

Ambagon Therapeutics, another molecular glue drug discovery company, applies a new approach to a challenging target class, the intrinsically disordered proteins. It involves forcing disordered proteins to acquire a druggable interface using molecular glues to stabilize their interaction with 14–3–3 adaptor proteins, a signaling hub for critical cell processes. It recently announced an ambitious program to augment its drug discovery platform and to advance its pipeline of molecular glues.^124^ Their pipeline currently focuses on oncology, offering many opportunities to engage currently undruggable targets. The company expects to announce at least one development candidate in 2023 and to enter the clinic in 2024.^125^

**Figure 11:**
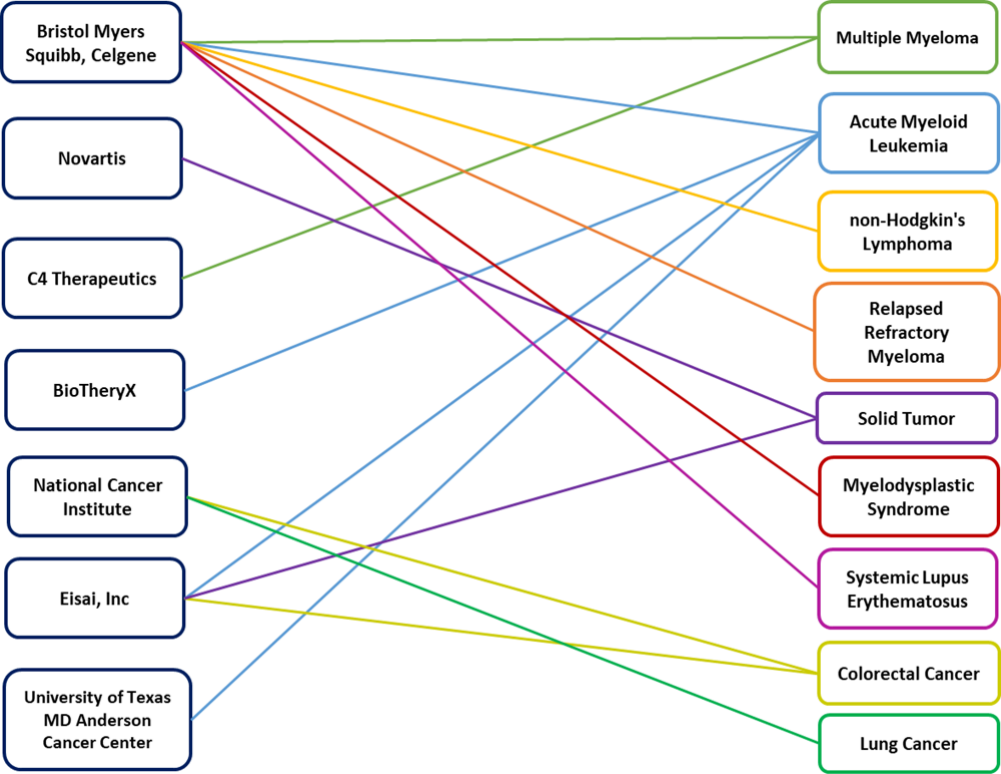
Companies and research organizations with discovered molecular glues in the clinical developmental pipeline and the diseases they treat (Table S1).

Several other companies highlighted in Figure 11 all have molecular glue compounds in various stages of clinical development (Table S1), treating many different solid and liquid tumors along with inflammatory conditions and autoimmune disease such as systemic lupus erythematosus.

Bristol Myers Squibb (New York, NY) is a company developing molecular glue compounds that have progressed to the market. Molecular glue therapeutics Pomalyst (pomalidomide), which is indicated in the treatment of multiple myeloma,^126^ and Revlimid (lenalidomide), for the treatment of multiple myeloma, myelodysplastic syndrome, and mantle cell lymphoma,^127^ were approved by the U.S. FDA in 2013 and 2017, respectively. Novartis (Basel, Switzerland) is another company with an approved molecular glue. Mekinist (trametinib), currently on the market for the treatment of melanoma, was approved by the U.S. FDA in 2017.

### Noteworthy Patents

There are a growing number of patents related to molecular glues in the CAS Content Collection. Most of them provide large libraries of compounds along with their synthesis routes, as well as *in vivo* and *in vitro* testing results. Listed in Table 12 are some noteworthy molecular glue-related patents.

**Table 12:** Notable Molecular Glue-Related Patents

patent number	title	organization	highlights
WO 2021/053555	glue degraders and methods of use thereof	Novartis AG	glue degrader compounds binding to and altering the specificity of a cereblon (CRBN) complex to induce ubiquitination and degradation of a protein; binders to the tris-tryptophan pocket of cereblon E3 ligase; 18 compounds synthesized and tested
WO 2021/249517	a molecular glue regulating CDK12–DDB1 interaction to trigger cyclin K degradation	National Institute of Biological Sciences, Beijing	molecular glues for triggering polyubiquitination and degradation of CCNK (cyclin K); creating a modified CDK12 protein binding DDB1 of the DDB1–CUL4–RBX1 complex; 31 compounds synthesized and tested
WO 2020/006264	ligands to cereblon (CRBN)	Dana-Farber Cancer Institute	compounds with immunomodulatory activity, methods of making them, and pharmaceutical compositions; 61 compounds synthesized and tested; ∼70 potential targeted proteins listed
WO 2008/115516	4′-O-substituted isoindoline derivatives and compositions comprising and methods of using the same	Celgene Corp.	74 4′-O-substituted isoindoline derivative compounds synthesized and tested; pharmaceutical compositions of these compounds disclosed
WO 2021/126805	modulation of protein degradation	Orionis Biosciences	agent in treating a disease by recruitment and/or ubiquitination and/or degradation of proteins such as argininosuccinate synthetase
WO 2021/178920	compounds for targeted degradation of BRD9	C4 Therapeutics	BRD9 protein degradation compounds provided for treatment of disorders mediated by BRD9, including abnormal cellular proliferation
WO 2021/127080	detection of novel degradation-related interactions	Orionis Biosciences	method for detecting and identifying protein–protein or protein–small molecule interactions using a MAPPITT assay with CRBN, IKZF1, DDB1, PROTAC, FKBP1A, and VHL
WO 2014/094138	screening methods to identify inhibitors of E2 enzymes by stabilization of noncovalent ubiquitin–E2 complexes for use in cancer therapy and other disorders	University of Montreal	stabilization of the interaction of noncovalent donor ubiquitin with E2 enzymes, including CDC34–ubiquitin interaction, and therapeutic methods for inhibiting enzymes involved in the cell ubiquitin–proteasome system (UPS)
WO 2015/200795, WO 2017/117118	compositions and methods for inducing conformational changes in cereblon other E3 ubiquitin ligases	Celgene Corp.	screening methods, computational methods, and biomarkers based on the elucidation of the interaction among cereblon, its substrates, and certain compounds or agents, including small molecules, peptides, and proteins
WO 2020/079103	method for identifying a chemical compound or agent inducing ubiquitination of a protein of interest	CEMM Research Center for Molecular Medicine	method for identifying compounds or agents that can induce ubiquitination of a protein of interest, for treating cancer or other diseases

## Summary and Outlook

Proximity-induced protein degradation using targeted degraders has emerged recently as a favorable approach in drug discovery and development. In one of the approaches, E3 ligases are being reprogrammed by monovalent small molecules, termed molecular glues. Binding of the ligand to the E3 ligase modifies the properties of protein interface, leading to dimerization with a neosubstrate. It has become clear lately that molecular glue type binding can be considered as a new modality option, specifically for otherwise poorly druggable protein targets. In this way, inducing protein degradation via small molecules has become a favorable therapeutic paradigm.

Altogether, only ∼16% of the disease-related proteins have been targeted by a drug (small molecule or biologic) today.^145^ Estimates show that ∼2% of the rest have been successfully knocked down by TPD in recent years.^146^ Considering the relatively new and rapid emergence of TPD as a protein knockdown strategy, the percentage of successfully degraded targets seems rather impressive and is expected to further increase.

Although the human genome encodes more than 600 E3 ligases, very few of them (VHL, CRBN, IAP, and MDM2) have been used to trigger small molecule-induced degradation. Because different ligases show specificity for recruitment and degradation of unique target proteins, expanding the reach of molecular glue degraders and eliminating so far undruggable proteins by accurately selecting new E3 ligases as targets for drug discovery are believed to be possible. Moving beyond the most widely employed cereblon E3 ligase will therefore significantly expand the therapeutic capacity of induced protein degradation.

Despite the favorable pharmacokinetic properties of molecular glues, currently well-characterized molecular glues are limited. The understanding of their mode of action and design principles is still deficient; thus, advanced research in the area is highly desirable. Advancement of the proximity-based platforms will be highly impactful for drug discovery. For instance, many disease-relevant undruggable proteins are known for having regions of intrinsic disorder^147^ that cannot be targeted by conventional small molecule drugs, whereas molecular glues can make a significant impact. In binding to a disordered protein region, molecular glues induce order, thus conferring druggability. Therefore, careful screening using innovative approaches may lead to the discovery of ligandable pockets in a variety of undruggable targets and could open the possibility of targeting intrinsically disordered regions of proteins, particularly such with a high degree of disorder, including transcription factors, adaptor/scaffolding proteins, and RNA binding proteins.^53,124^

Since the initial serendipitous findings of molecular glues, a bottleneck setback has been how to efficiently approach molecular glue discovery and design. The currently applied methods mostly rely on intensive high-throughput chemical screening, followed by systematic validation and lead optimization. The emerging development of efficient rational discovery strategies and structure-based drug development pipelines is enhancing the efficiency and applicability of molecular glue discovery. Despite the progress in protein science, a profound understanding of such compound-mediated protein–protein binding events is broadly insufficient. Detailed knowledge of the interfaces involved, as in the example of IMiDs binding to CRBN, is necessary to design novel molecular glues and develop them into a powerful new medicines.

The development of new computational tools, such as molecular docking tools, that model and foretell the binding mode of molecular glue-induced PPI complexes is proving to be a valuable advancement for identifying novel compatible E3 ligase–target protein partners through virtual screening and in the structure-based rational design of new optimized molecular glues. However, the application of these tools depends on a thorough structural understanding of the chemistry at the dimer interface and how a molecular glue stabilizes dimerization with the target protein, while reducing off-target binding. Moreover, a better understanding of the minimal degrons necessary for PPI and small molecule protein interactions at the interface can inform the development of artificial intelligence technologies beneficial for upgrading the efficiency of data mining and molecular design.

As small molecule drugs, one of the hurdles in molecular glue advancement is their progression into clinical therapeutics. Although some progress in understanding their mechanisms of action has been achieved, the pharmacokinetic and pharmacodynamic profiles of newly developed molecular glues are still largely unknown, which obstructs their further development into drug candidates. Comprehensive studies of the detailed pathways of pathogenic protein degradation via the ubiquitin–proteasome system enabled by each specific molecular glue are greatly important for their optimization and perfection into successful drugs. Despite these hurdles, molecular glue companies and research organizations are taking on the challenge to progress discovered molecular glues to the clinic and ultimately to market. From established companies and research organizations to 2022 start-up companies, they all have the same mission to treat diseases with high unmet needs and transform how disease treatment is approached.

In addition to proteasomes, lysosomes provide another independent pathway for the eukaryotic cells to degrade disease-related proteins. Recently, targeted protein degradation strategies via the lysosomal pathway have been explored that also could degrade membrane proteins, extracellular proteins, and protein aggregates, thus greatly expanding the range of substrates for TPD.^148^ As a result of intensive research in the area, a number of new strategies via the lysosomal pathway, such as LYTAC, AbTAC, ATTEC, AUTAC, bispecific aptamer chimeras, and AUTOTAC, have recently emerged.^149^

In the long run, structure-based rational optimization approaches for perfection of the targeted protein degraders are urgently needed. Altogether, advanced knowledge of the precise mechanisms of the operation of molecular glues and their structural biology and medicinal chemistry features would be of utmost importance in transforming and progressing the targeted protein degradation strategy into a favorable practical application in the clinic.

## Supporting Information Available

ChemScape map of compounds within the 90% similarity limit with respect to thalidomide used as protein degraders according to SciFinder^n^ (Figure S1), bioactivity indicators of the 310 compounds within the 85–99% chemical structure similarity range with respect to the molecular glue CC-885 as estimated using SciFinder^n^ (Figure S2), and bioactivity indicators of 715 compounds with an α,α-difluorobenzeneacetamide CC-885 analogue chemical substructure as estimated using SciFinder^n^ (Figure S3) (PDF)
E3 ligase- and non-E3 ligase-based molecular glue degraders (Table S1) (XLSX)

## Abbreviations

AR: androgen receptor
ASB6: ankyrin repeat and SOCS box containing 6
ATTEC: autophagosome-tethering compound
AUX: auxin
BCL6: B-cell lymphoma 6
CDC34: ubiquitin-conjugating enzyme E2 R1
CELMoD: cereblon E3 ligase modulating drug
CEP250: centrosomal protein 250
cIAP: cellular inhibitor of apoptosis protein 1
CNS: central nervous system
CRBN: cereblon
CsA: cyclosporin A
DCAF: DDB1- and CUL4-associated factor
DDB1: DNA damage binding protein 1
DLBCL: diffuse large B-cell lymphoma
ER: estrogen receptor
FBXO: F box only protein
FKBP: FK506 binding protein
IKZF1: Ikaros
IKZF2: Helios
IKZF3: Aiolos
HDAC: histone deacetylase
IMiDs: immunomodulatory imide drugs
JA: jasmonate
JAZ: jasmonate-ZIM
KEAP1: Kelch-like ECH-associated protein 1
MAX: Myc-associated factor X
MDM2: mouse double minute 2 homologue
mHTT: mutated huntingtin
mTOR: mammalian target of rapamycin
NSCLC: non-small cell lung cancer
PI3K: phosphoinositide
POI: protein of interest
polyQ: polyglutamine
PPI: protein–protein interaction
PROTAC: proteolysis targeting chimera
RBM39: RNA binding motif protein 39
RNF: RING finger protein
SALL4: Sal-like protein 4
SBDD: structure-based drug design
SCF: Skp1-cullin 1-F box
SfA: sanflifehrin A
SIAH1: seven in absentia homologue 1
SKP: S phase kinase-associated protein
STUB1: STIP-1 homology and U box-containing protein 1
TPD: targeted protein degrader
β-TRCP: β-transducin repeat-containing protein
UBE4A: E3/E4 ubiquitin ligase
UPS: ubiquitin–proteasome system
URB2: unhealthy ribosome biogenesis protein 2
VHL: von Hippel–Lindau
WIPO: World Intellectual Property Organization.
