# Supplementary material for: Molecular Glues: The Adhesive Connecting Targeted Protein Degradation to the Clinic
Source: Biochemistry. 2022 Jul 20;62(3):601–23. doi: 10.1021/acs.biochem.2c00245 (PMC9910052; doi:10.1021/acs.biochem.2c00245)
Supplement: Supplementary file 1 — bi2c00245_si_001.pdf [file bi2c00245_si_001.pdf]

Supporting Information

Molecular glues: The adhesive connecting targeted protein degradation to the clinic

Janet M. Sasso, Rumiana Tenchov, DaSheng Wang, Linda S. Johnson, Xinmei Wang, Qiongqiong Angela Zhou*

CAS, a division of the American Chemical Society, 2540 Olentangy River Rd, Columbus, OH 43202, USA

Contents:

Figure S1. ChemScape map of compounds within 90% similarity limit to thalidomide used as protein degraders according to SciFinder^n.

Figure S2. Bioactivity Indicators of the 310 compounds within the 85% - 99% chemical structure similarity range to the molecular glue CC-885 as estimated using SciFinder^n.

Figure S3. Bioactivity Indicators of 715 Compounds with an α,α-Difluorobenzeneacetamide CC-885 analog chemical substructure as estimated using SciFinder^n.

Chemscape map of compounds within 90% similarity limit to thalidomide used as protein degraders (SciFinder^{n})

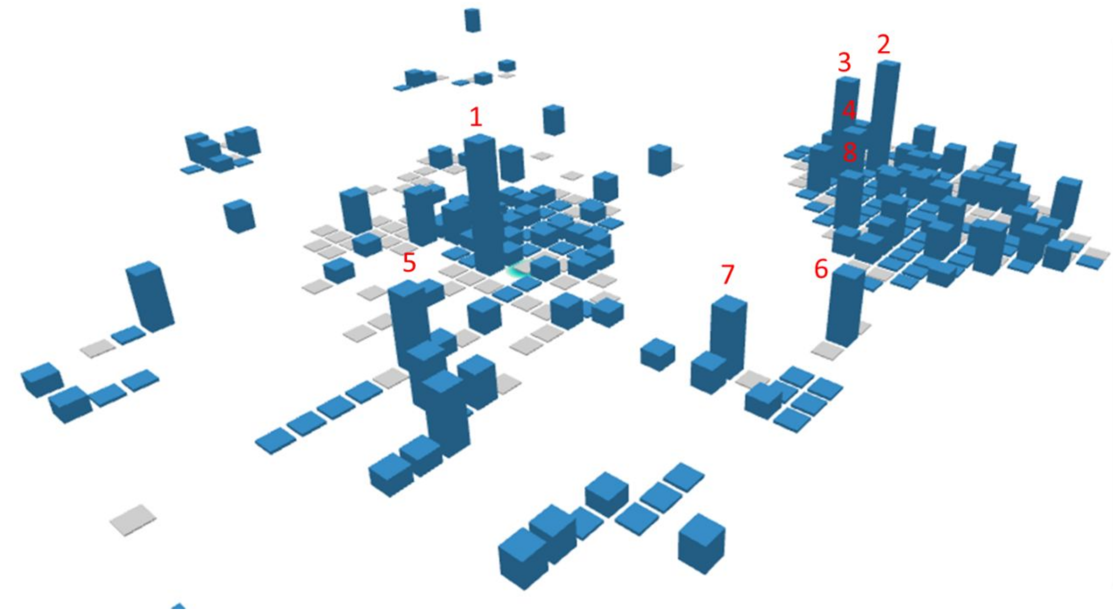

	Compound CAS #	Structure	Number of patents
1	50-35-1 (thalidomide)	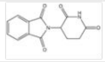	1,060
2	191732-72-6 (lenalidomide)	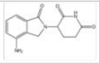	962
3	19171-19-8 (pomalidomide)	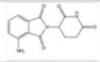	496
4	5054-59-1	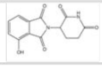	104
5	64567-60-8	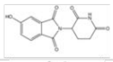	39
6	1061604-41-8	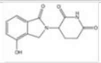	28
7	191732-76-0	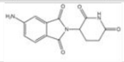	24
8	191732-70-4	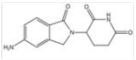	23

Figure S1. A search in SciFinder^{n} for compounds within the 90% similarity limit to thalidomide used as protein degraders gave 219 compounds. Each column on the figure reflects a single compound, with the top 8 indicated by numbers. The distance between two columns reflects the similarity between these two compounds. The bar height reflects the number of patents related to a given compound. The table on the right lists the top 8 compounds of this collection.

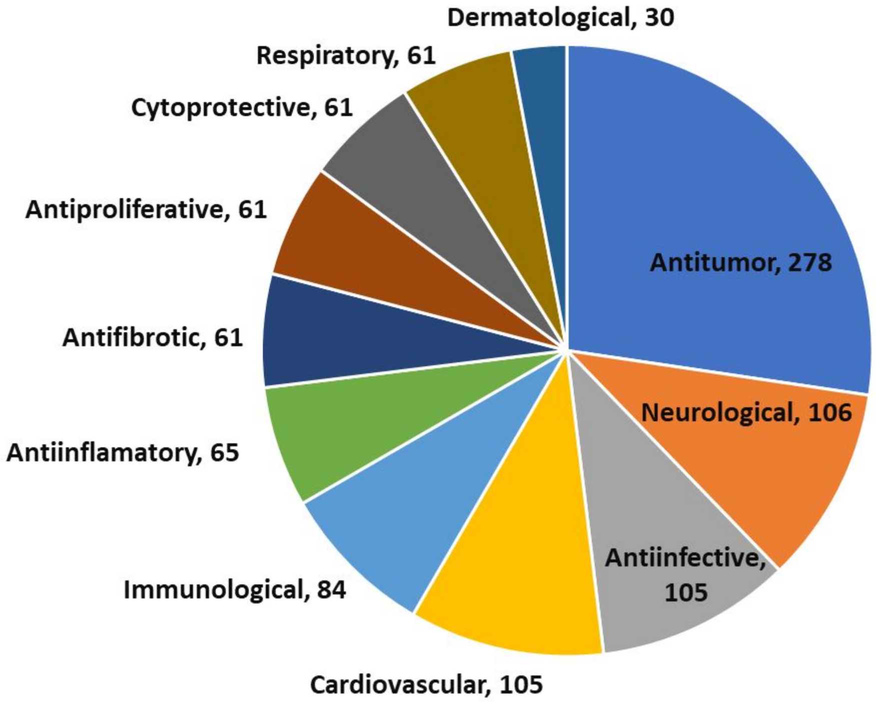

Figure S2. Bioactivity Indicators of the 310 compounds within the 85% - 99% chemical structure similarity range to the molecular glue CC-885 as estimated using SciFinder^{n}. The bioactivity indicators are common for multiple compounds.

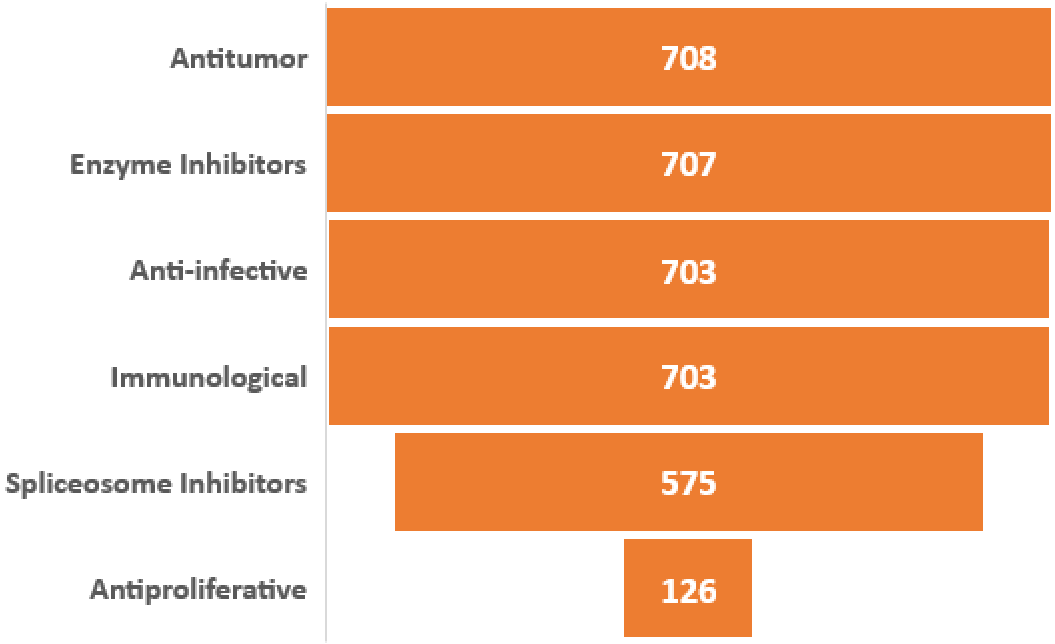

Figure S3. Bioactivity Indicators of the 715 Compounds with an α,α-Difluorobenzeneacetamide CC-885 analog chemical substructure: 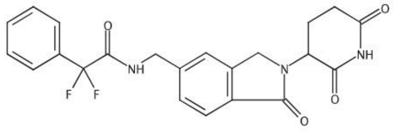 as estimated using SciFinder^{n}. The bioactivity indicators are common for multiple compounds.
